# Conjugated oligoelectrolytes overcome cancer drug resistance by dual-mode lysosomal membrane disruption

**DOI:** 10.20517/cdr.2025.196

**Published:** 2026-03-20

**Authors:** Lingna Wang, Yufei Mao, Yu Dong, Manqi Tan, Xingyu Wang, Zhaobo Liu, Chenyao Nie, Shu Xing, Meng Li, Haitao Yuan, Bing Wang

**Affiliations:** ^1^Ningbo Institute for Drug Control, Ningbo 315048, Zhejiang, China.; ^2^Laboratory of Advanced Theranostic Materials and Technology, Ningbo Institute of Materials Technology and Engineering, Chinese Academy of Sciences, Ningbo 315201, Zhejiang, China.; ^3^Ningbo Cixi Institute of Biomedical Engineering, Ningbo 315300, Zhejiang, China.; ^4^Cixi Biomedical Research Institute, Wenzhou Medical University, Wenzhou 315302, Zhejiang, China.; ^5^College of Pharmaceutical Science, Zhejiang University of Technology, Hangzhou 310014, Zhejiang, China.; ^6^Center for Drug Research and Development, Guangdong Provincial Key Laboratory for Research and Evaluation of Pharmaceutical Preparations, Guangdong Pharmaceutical University, Guangzhou 510006, Guangdong, China.

**Keywords:** Membrane-intercalating conjugated oligoelectrolytes, lysosomal membrane permeabilization, drug resistance, combination therapy

## Abstract

**Aim:** Multidrug resistance (MDR) often arises from lysosomal sequestration of chemotherapeutics. This study aims to design and evaluate lysosome-targeting membrane-intercalating conjugated oligoelectrolytes (MICOEs) for their potential to reverse MDR via dual-mode lysosomal membrane disruption, and to identify the most effective candidate.

**Methods:** Three MICOEs featuring a pyridothiadiazole-thienothiophene-pyridothiadiazole (PTTP) conjugated backbone with quaternary ammonium-terminated 4-, 6-, and 8-carbon alkyl chains at both ends (PTTP-DC4, PTTP-DC6, PTTP-DC8) were synthesized and characterized. Their photophysical properties, cellular uptake, and sublocalization were assessed in doxorubicin (DOX)-resistant Michigan Cancer Foundation-7/adriamycin-resistant (MCF-7/ADR) cells. Lysosomal integrity and contents release were evaluated via acridine orange and cathepsin B assays. Proteomic analysis was performed to uncover mechanisms. The combinational effect of PTTP-DC6 and DOX was tested in drug-resistant two-dimensional (2D) and three-dimensional (3D) cell models.

**Results:** Among PTTP-DCns (where n = 4, 6, and 8, corresponding to PTTP-DC4, PTTP-DC6, and PTTP-DC8), PTTP-DC6 showed optimal lysosomal accumulation and induced lysosomal membrane permeabilization (LMP) through both physical membrane interaction and light-triggered reactive oxygen species generation. Proteomic analysis revealed significant enrichment of pathways associated with oxidative stress and lysosomal dysfunction. Pretreatment with PTTP-DC6 at low doses, particularly under mild light irradiation, significantly enhanced DOX sensitivity in resistant 2D monolayers and 3D spheroid models.

**Conclusion:** PTTP-DC6 overcomes MDR by dual-mode LMP induction, providing a simple strategy to resensitize resistant cancers to conventional chemotherapy.

## INTRODUCTION

Chemotherapy stands as a pivotal tool in the arsenal of cancer therapeutics, particularly for mid-to-late-stage tumors with high metastatic potential^[1]^. However, the emergence of multidrug resistance (MDR) in tumor cells, which contributes to over 90% cancer-related fatalities, remains a significant challenge in cancer therapy^[2-4]^. This underscores the urgent need to address this persistent issue to improve patient survival outcomes.

One key role in cancer drug resistance is the lysosome^[5-9]^, which not only degrades chemotherapeutic agents through enzymatic hydrolysis but also sequesters them away from their intracellular targets, effectively reducing the active drug concentration^[10,11]^. Lysosomal drug sequestration is particularly relevant for weak-base chemotherapeutics - such as doxorubicin (DOX) - through ion trapping, thereby impairing drug access to nuclear targets and promoting resistance^[12]^. To overcome this, strategies inducing lysosomal membrane permeabilization (LMP) have been developed, primarily through either physical membrane disruption (e.g., *in-situ* self-assembling peptides, cationic amphiphilic drugs)^[13-15]^ or reactive oxygen species (ROS)-mediated chemical degradation (e.g., ferroptosis inducers, nanozymes)^[16-18]^. However, many of these approaches lack specific lysosome membrane targeting ability, and rely on single mechanisms or complex nanomaterials^[19,20]^, which may limit their efficacy and translational potential. Thus, designing a simple molecular material capable of lysosome membrane targeting for dual-mode function modulation is particularly crucial.

Membrane-intercalating conjugated oligoelectrolytes (MICOEs) emerge as an ideal candidate to tackle this challenge. Characterized by a linear π-conjugated backbone and positively charged side chains at both ends, MICOEs can form stable assemblies with lipid membranes through synergistic hydrophobic and electrostatic interactions^[21,22]^. This intercalation can be utilized to finely tune cell membrane permeability to small molecules by adjusting the distance between the charges of MICOEs^[23-26]^. Furthermore, the conjugated backbone endows MICOEs with excellent photoactivity, enabling efficient light-induced *in-situ* membrane disruption^[27,28]^. This unique combination of membrane-embedding capability and light-induced reactivity makes MICOEs a quintessential molecular platform for dual-mode membrane modulation.

**Scheme 1:**
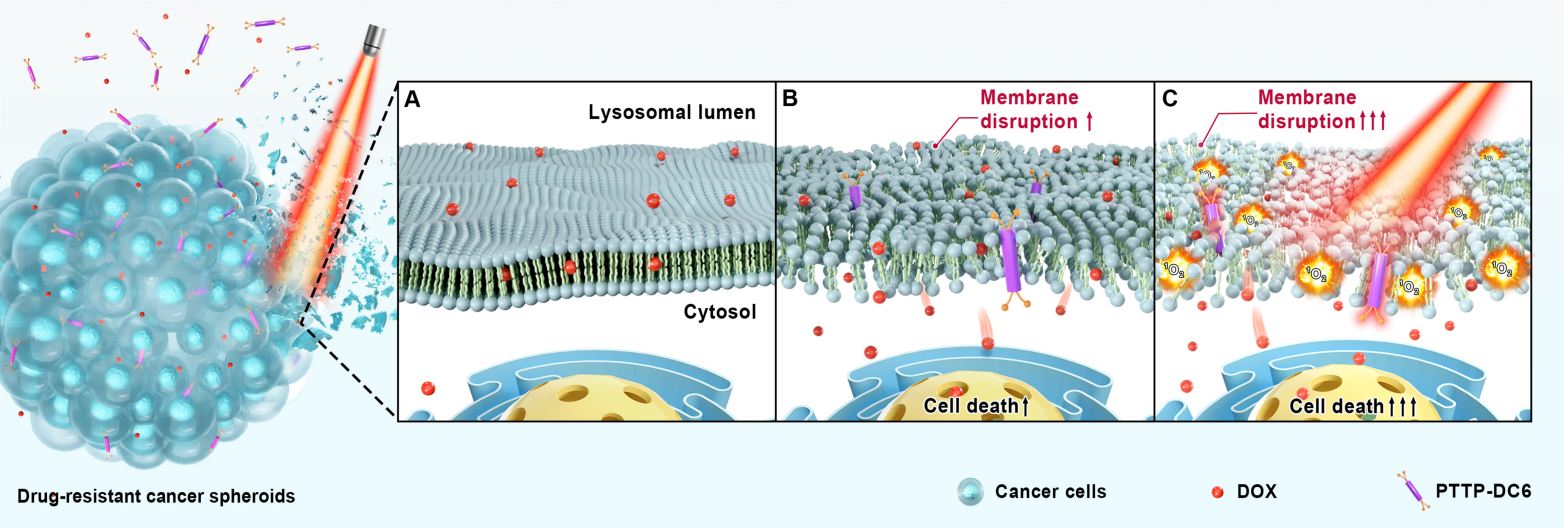
Schematic illustration of the mechanism by which PTTP-DC6 overcomes drug resistance in tumor spheroids through dual-mode membrane disruption. Created in Blender. (A) The dysfunctional membranes in DOX-resistant cancer cells prevent DOX from accumulating effectively in the nucleus; (B) In the dark, membrane insertion by PTTP-DC6 induces membrane disruption, enhancing the sensitivity of MDR cells to DOX; (C) Upon light irradiation, PTTP-DC6 generates ^1^O_2_ *in situ*, which further disrupts lysosome membranes, and leads to significantly improved therapeutic outcomes. PTTP-DC6: Benzene-pyridothiadiazole-thienothiophene-pyridothiadiazole-benzene conjugated framework with quaternary ammonium-terminated C6 alkyl chains at both ends; DOX: doxorubicin; MDR: multidrug resistance.

With these points in mind, we developed a novel lysosome-targeting MICOE photosensitizer, PTTP-DC6, to recover anticancer activity of a conventional chemotherapeutic drug (DOX) in MDR cancer cells. As illustrated in Scheme 1, PTTP-DC6 can intercalate into the lysosomal membrane, augmenting membrane permeability through its intrinsic capability of membrane regulation in the dark [Scheme 1B]. Under light exposure, PTTP-DC6 can further generate singlet oxygen (^1^O_2_), inducing localized lipid peroxidation and amplifying membrane disruption [Scheme 1C]. This dual physical and oxidative action synergistically promotes LMP, facilitating the release of trapped DOX molecules and restoring drug efficacy. In this study, we demonstrate that this simple molecular system enables highly effective reversal of tumor MDR at low doses, offering a promising strategy to combat chemoresistance.

## METHODS

### Preparation of liposomes (PTTP-DCn@Ls)

A 1.8:1:0.2 molar ratio of DSPC:cholesterol:DSPE-PEG2000 was used to prepare blank liposomes. The lipid materials (10 mg) were dissolved in a chloroform-methanol mixture (4:1, v/v). The organic solvent was subsequently removed under reduced pressure at 55 °C to form a thin lipid film. This film was then hydrated with 5 mL of pure water or solutions of PTTP-DCns (100 μM in water) to prepare uniform liposomes via sonication and extrusion at 55 °C. The resulting liposomes encapsulating PTTP-DCns were designated as PTTP-DCn@Ls.

### Spectral measurements

Stock solutions of PTTP-DCns (1 mM) were prepared with water, which were diluted to the working solutions (10 μM) with 1x phosphate-buffered saline (PBS), dimethyl sulfoxide (DMSO), and methanol (MeOH), respectively. The absorption spectra of PTTP-DCns in different solvents were recorded with a ultraviolet-visible (UV-Vis) spectrometer, while the fluorescence spectra of each solution were recorded with a fluorescence spectrometer. The excitation wavelength was 520 nm, and the fluorescence emission signals were collected from 550 to 900 nm.

### Singlet oxygen generation

For the ^1^O_2_ measurement using singlet oxygen sensor green (SOSG), an SOSG stock solution (5 mM in methanol) was diluted to 200 μM with 1x PBS. Solutions of Rose Bengal (RB), methylene blue (MB), PTTP-DCns, and PTTP-DCn@Ls were prepared in PBS at a concentration of 2.5 μM. Then, 195 μL of each sample was mixed with SOSG (final concentration: 5 μM) in a black 96-well plate. The mixtures were irradiated under white light (equipped with a UV cut filter, 10 mW·cm^-2^) for 10 min, and fluorescence emission at 525 nm was monitored, with an excitation wavelength (λ_ex_) of 488 nm.

For the quantitative determination of singlet oxygen quantum yield (*Φ*_Δ_) via ^1^O_2_ phosphorescence detection, PTTP-DCns and RB were dissolved in deuterated PBS and adjusted to the same absorbance at 520 nm (A = 0.7). The solutions were degassed by nitrogen bubbling for 10 min. The near-infrared emission between 1,200-1,400 nm was collected. A fourth-order polynomial fitting method was used to fit the data of degassed PTTP-DCns from 1,200-1,230 nm and 1,310-1,370 nm. The fitting curve was used to simulate the emission spectrum of PTTP-DCns as background to get the ^1^O_2_ phosphorescence spectrum in PTTP-DCns solution. Then, the ^1^O_2_ phosphorescence spectra were fitted by a GaussAmp fitting method in the origin software. The ^1^O_2_ quantum yield (*Φ*_Δ_) can also be calculated as^[27]^:

**Figure:**
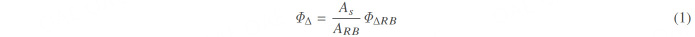


where *A_s_* and *A_RB_* are the intensity of ^1^O_2_ phosphorescence generated from photosensitization of testing samples and RB, respectively, by calculating the integral area of the final GaussAmp fitting curve. *Φ*_Δ_*_RB_* (0.76) is used as the ^1^O_2_ quantum yield of RB in PBS buffer.

### Cell lines and cell incubation conditions

Michigan Cancer Foundation-7/adriamycin-resistant (MCF-7/ADR, DOX-resistant human breast cancer cell line) cells were obtained from Shanghai Jinyuan Biotechnology Co., Ltd. (China) and cultured in Roswell Park Memorial Institute (RPMI) 1640 medium supplemented with 10% fetal bovine serum (FBS), 1% penicillin-streptomycin (P/S) and 0.5 μg·mL^-1^ DOX.

HCC827/IR (icotinib-resistant human lung cancer cell line) cells were obtained from Ningbo University Health Science Center (China) and cultured in RPMI 1640 medium supplemented with 10% FBS, 1% P/S and 10 μM icotinib.

HEK293 (human kidney cell line) cells were obtained from Wuhan Pricella Biotechnology Co., Ltd (China) and cultured in high-glucose Dulbecco’s Modified Eagle Medium (DMEM) supplemented with 10% FBS and 1% P/S.

Cells were all cultured at 37 °C in a humidified incubator containing 5% (v/v) CO_2_.

### Cellular uptake

To reduce the fluorescence interference of DOX, MCF-7/ADR cells were cultured without DOX for 2-3 generations before confocal laser scanning microscope (CLSM) imaging.

The cellular uptake of the PTTP-DCns compounds in MCF-7/ADR cells was assessed under both short-term and long-term conditions. For all experiments, cells were treated with 1 µM of the specified compound in serum-supplemented medium. For short-term monitoring, cells treated for 2 h were analyzed by flow cytometry. For long-term tracking, CLSM was performed at intervals from 15 min to 24 h post-treatment without prior washing.

### Cellular uptake inhibition

Following adhesion for 12 h, the MCF-7/ADR cells were pre-treated for 30 min under different conditions: either with serum- and phenol red-free medium at 4 °C, or with the same medium containing specific inhibitors [chlorpromazine (CPZ) at 10 µg·mL^-1^, genistein (Gen) at 50 µg·mL^-1^, or dynasore (Dyn) at 80 µM] at 37 °C. PTTP-DCns were then added to each dish at a final concentration of 1 μM, followed by 4 h of continued incubation. The cells were subsequently analyzed directly by CLSM imaging.

### Cellular distribution

Following overnight adhesion in glass-bottom dishes (1 × 10^5^ cells/dish), the MCF-7/ADR cells were incubated with PTTP-DCns (1 μM) for 24 h after medium removal, and subsequently treated with Lyso-Tracker Green DND-26 (5 µM) for 1 h. After being washed twice with PBS buffer containing 1% FBS, the cell samples were imaged with a high-speed CLSM. Lyso-Tracker Green DND-26 (excited at 488 nm, emission 500-540 nm) was displayed in green, and PTTP-DCns (excited at 552 nm, emission 660-740 nm) in red.

### Cell viability assay

For the cytotoxicity of PTTP-DCns and chloroquine (CQ), MCF-7/ADR or HEK293 cells were seeded in 96-well plates and incubated for 24 h at 37 °C. The medium was then replaced with 0-25 µM of PTTP-DC4, PTTP-DC6, PTTP-DC8 or 0-10 µM of CQ. After another 24 h, light-treated groups were irradiated with 525 nm light emitting diode (LED) light (0.2 mW·cm^-2^, 30 min); the rest remained in the dark. All Cells were cultured in the incubator for an additional 24 h.

To evaluate the effect of PTTP-DCns on drug sensitivity, MCF-7/ADR and HCC827/IR cells were treated with a fixed concentration (1 µM) of each compound for 24 h, followed by the same light/dark protocol described above. After irradiation, the medium was changed to a series of DOX solutions (0-100 µg·mL^-1^) or icotinib solution (0-25 µM), and cells were cultured for an additional 48 h.

To evaluate the effect of CQ on drug sensitivity, MCF-7/ADR cells were treated with 1 µM of CQ for 24 h, followed by change of culture medium with 100 μg·mL^-1^ DOX and an additional incubation of 48 h.

Viability was assessed using the 3-(4,5-dimethylthiazol-2-yl)-2,5-diphenyltetrazolium bromide (MTT) assay. Absorbance was measured at 570/600 nm. All experiments were performed in triplicate.

### Cellular ROS level

Following a 24-hour treatment with or without 1 μM PTTP-DC6, MCF-7/ADR cells were incubated with 10 μM 2′,7′-dichlorodihydrofluorescein diacetate (DCFH-DA) for 1 h at 37 °C. After two washes with PBS containing 1% FBS, the dishes were filled with serum- and phenol red-free medium for subsequent analysis. The dishes of light groups were irradiated with a 525 nm LED light at an intensity of 0.2 mW·cm^-2^ for 30 min, while the rest were kept in the dark at room temperature. Thereafter, the cells were directly analyzed via CLSM imaging. ROS generation was monitored by measuring the fluorescence of the oxidation product (2,7-dichlorofluorescein, DCF), which was excited at 488 nm with emission collected in the 500-550 nm range.

### AO redistribution assay

Following overnight adhesion in glass-bottom dishes (1 × 10^5^ cells/dish), the MCF-7/ADR cells were incubated with blank medium or PTTP-DCns (1 μM) for 24 h, and the dishes of light groups were irradiated with a 525 nm LED light (0.2 mW·cm^-2^, 30 min), while the rest were kept in the dark at room temperature. Then the cells were incubated with acridine orange hydrochloride hydrate (AO, 5 μg/mL) for 15 min at 37 °C and washed twice with PBS buffer. Fluorescence images were captured with CLSM (λ_ex_ = 488 nm), with emission collected in the green channel (λ_m,green_ = 500-550 nm) and red channel (λ_m,red_ = 650-710 nm).

### Detection of cathepsin B release

The pretreatment of MCF-7/ADR cells was consistent with that of AO redistribution assay. Cells were incubated in blank medium or PTTP-DC6 (1 μM) solution for 24 h, then irradiated at 525 nm (0.2 mW·cm^-2^) for 30 min or darkened, washed twice with PBS buffer, incubated with cathepsin B substrate (Green Cathepsin B Assay Kit) for 1 h, washed twice with PBS, as observed by CLSM. λ_ex_/λ_em_: cathepsin B (green) was 488/500-540 nm, PTTP-DC6 (red) was 552/600-740 nm.

### Pro DIA quantitative proteomics assay

MCF-7/ADR cells were cultured in T175 tissue culture flasks (culture area: 175 cm^2^) and divided into three experimental groups (*n* = 3): a blank control, a DC6-treated group, and a DC6-treated group with light exposure (DC6-L). When cell density reached approximately 60%, cells were treated with either blank medium or PTTP-DC6 (1 μM) solution for 24 h, then irradiated at 525 nm (0.2 mW·cm^-2^) for 30 min or darkened. Following a subsequent 1-hour incubation, cells were harvested and subjected to quantitative proteomics analysis at Shanghai OE Biotech Co., Ltd. (China).

Data-independent acquisition mass spectrometry (DIA-MS) raw data were processed using DIA-NN (an automated software suite for DIA proteomics data processing, version V2.3.2) for protein identification and quantification. Proteins with excessive missing values across samples and those lacking unique peptides were excluded. Missing values were imputed using the group mean or half of the minimum value in the expression matrix. The data were then normalized by median centering and log2-transformed. Differential expression analysis was performed using a *t*-test; proteins with a fold change ≥ 1.5 and a *P*-value < 0.05 were considered significant. Functional enrichment and gene set analysis of these differentially expressed proteins were conducted using clusterProfiler (version 4.9.1).

### 3D cytotoxicity assay

MCF-7/ADR spheroids were formed in the Akura™ PLUS Hanging Drop Plate and then transferred to the Akura™ 96 Spheroid Microplate. Following a recovery day (day 0), they were treated with 5 μM PTTP-DC6 or control medium for 24 h. One group was then irradiated with 525 nm light (1 mW·cm^-2^, 30 min), while another remained in the dark. All spheroids were subsequently exposed to 100 μg·mL^-1^ DOX for 48 h. Cell viability was assessed using the CellTiter-Glo® 3D assay. The cell viability rate of 3D microtissues (*VR_3D_*) was calculated as: *VR_3D_* = *L/L*_0_ × 100%, where *L* and *L*_0_ represent luminescence of treated and untreated control spheroids, respectively. All data were from four independent experiments. Meanwhile, the morphology of cell spheroids was recorded by a high-content imaging system on day 0 and day 3.

### Statistical analysis

Data are presented as mean ± standard deviation (SD). Student’s *t*-test was used to evaluate differences between two groups, whereas one-way analysis of variance (ANOVA) followed by Tukey’s post hoc test was applied for comparisons among three or more groups. Differences were deemed statistically significant when *P* < 0.05. All statistical tests were two-tailed. ^*^P < 0.05; ^**^P < 0.01; ^***^P < 0.001.

## RESULTS

### Preparation of PTTP-DCns and PTTP-DCn@Ls

**Figure 1:**
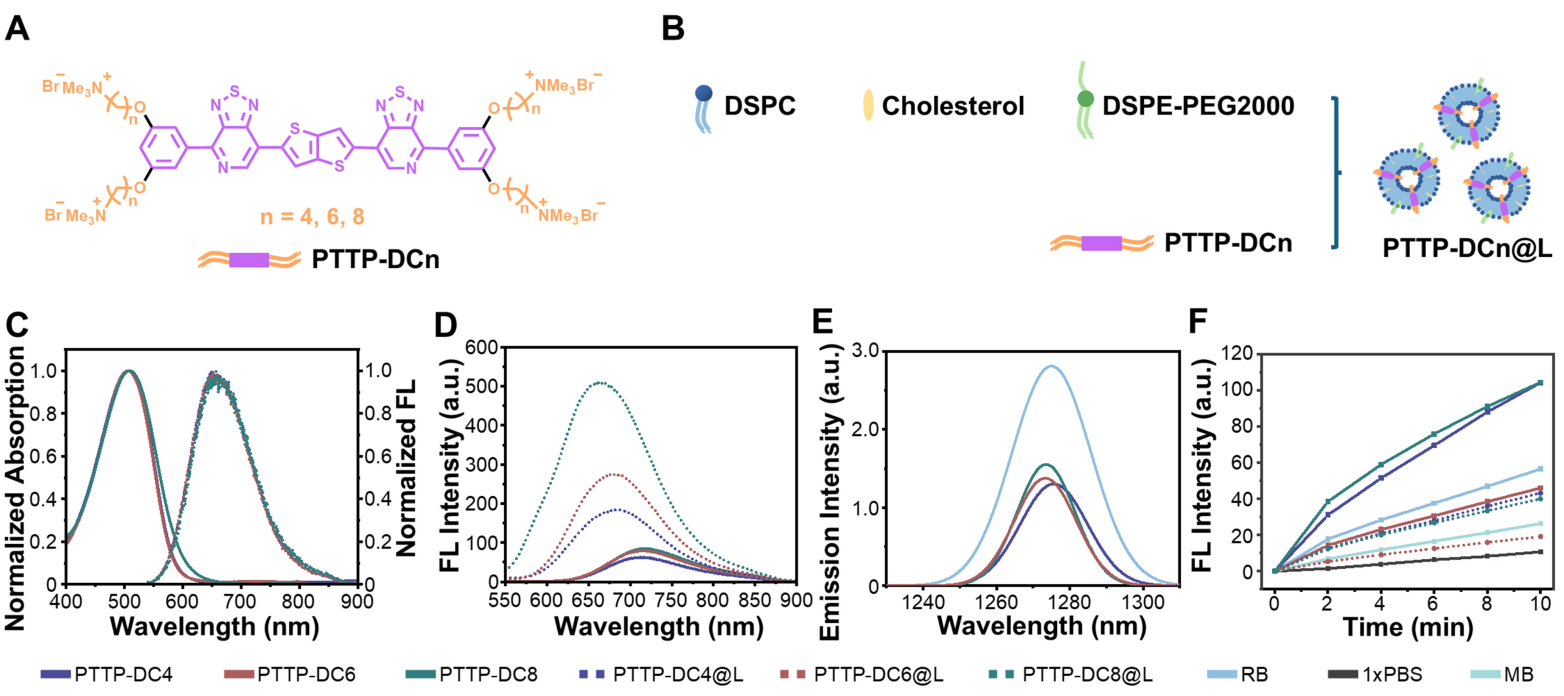
Preparation and optical properties of PTTP-DCns and PTTP-DCn@Ls. (A) Chemical structures of PTTP-DCns; (B) Composition diagram of PTTP-DCn@Ls; (C) Normalized absorption and fluorescence emission spectra (λ_ex_ = 520 nm) of PTTP-DCns in methanol; (D) Fluorescence emission spectra of 10 µM PTTP-DCns and PTTP-DCn@Ls in 1x PBS; (E) Phosphorescence emission spectra of ^1^O_2_ generated by PTTP-DCns and RB in deuterated PBS under 520 nm laser irradiation, and all the compounds have the same absorbance at 520 nm; (F) Fluorescence intensities of SOSG at 525 nm in the presence of PTTP-DCns, PTTP-DCn@Ls, RB, and MB after different irradiation time (white light, 10 mW·cm^-2^). PTTP-DCns: Benzene-pyridothiadiazole-thienothiophene-pyridothiadiazole-benzene conjugated framework with quaternary ammonium-terminated n-carbon alkyl chains at both ends; PBS: phosphate-buffered saline; RB: Rose Bengal; SOSG: singlet oxygen sensor green; MB: methylene blue; DSPC: 1,2-distearoyl-sn-glycero-3-phosphocholine; DSPE-PEG2000: 1,2-distearoyl-sn-glycero-3-phosphoethanolamine-N-[amino(polyethylene glycol)-2000] (ammonium salt); PTTP-DC4/6/8: benzene-pyridothiadiazole-thienothiophene-pyridothiadiazole-benzene conjugated framework with quaternary ammonium-terminated C4/C6/C8 alkyl chains at both ends.

As shown in Figure 1A, three PTTP-DCns with a shared conjugated backbone (highlighted in purple) and varying side chains of different lengths (indicated in orange) were designed and synthesized. The synthetic route of PTTP-DCns is depicted in Supplementary Scheme 1. Initially, the reaction between 1-bromo-3,5-dihydroxybenzene and alkyl dibromide with different carbon numbers generated end groups with varying side chains. Subsequent stannylation of end groups and two-step Stille coupling reactions with pyridinethiadiazole (PT) dibromide and bisstannyl thieno[3,2-b]thiophene, followed by a quaternization reaction, gave the final products PTTP-DC4, PTTP-DC6, and PTTP-DC8. Detailed synthesis procedures and spectra characterizations (proton nuclear magnetic resonance, ^1^H NMR, carbon-13 nuclear magnetic resonance, ^13^C NMR; high-resolution mass spectrometry, HRMS) of all intermediates and final products are provided in the Supplementary Materials.

To study the properties of PTTP-DCns in lipid bilayer environment, liposomes containing PTTP-DCns (noted as PTTP-DCn@Ls) were prepared via the thin film hydration method^[29-31]^, as depicted in Figure 1B and Supplementary Scheme 2. The hydrodynamic diameters of PTTP-DC4@L, PTTP-DC6@L, and PTTP-DC8@L were determined as 93.6 ± 2.0 nm, 87.5 ± 1.5 nm, and 110.5 ± 0.6 nm, respectively [Supplementary Figure 1]. Nevertheless, the zeta potentials increased gradually from 2.7 mV of blank liposome to 31.7 mV as side chains increased from C4 to C8 [Supplementary Figure 2].

### Photophysical and photochemical properties of PTTP-DCns

The absorption and fluorescence emission spectra of PTTP-DCns in solvents and lipids were first studied. In methanol, all three MICOEs showed similar maximum absorption peaks around 505 nm and maximum emission peaks around 650 nm [Figure 1C]. And significant red-shifts were observed on the absorption and fluorescence spectra of all PTTP-DCns as solvent polarity increased [Supplementary Figure 3], attributable to polar environment narrowing the band gap of intramolecular charge transfer (ICT) state^[32]^. Detailed photophysical parameters of PTTP-DCns and PTTP-DCn@Ls were summarized in Table 1. Notably, the absorption spectra of PTTP-DCns and PTTP-DCn@Ls in PBS (1x PBS, pH 7.4) were quite similar [Supplementary Figure 3 and Table 1], while remarkably enhanced and blue-shifted emissions were observed for PTTP-DCn@Ls [Figure 1D and Table 1]. The fluorescence quantum yield (*Φ_F_*) of PTTP-DC4, PTTP-DC6, and PTTP-DC8 increased to 2.2, 3.0, and 5.4 times, respectively, after insertion into liposomes, which may be attributed to the reduced quenching caused by the intermolecular aggregation and water after assembly with the membrane [Table 1 and Supplementary Figure 4].

**Table 1:** Absorption maxima (λ_abs_), molar extinction coefficients (ε), emission maxima (λ_em_), ^1^O_2_ quantum efficiencies (*Φ*_Δ_), and fluorescence quantum yields (*Φ_F_*) of PTTP-DCns and PTTP-DCn@Ls in 1x PBS

	**PTTP-DCns**			**PTTP-DCn@Ls**		
	**DC4**	**DC6**	**DC8**	**DC4**	**DC6**	**DC8**
**λ_abs_ [nm]**	518	522	521	522	525	523
**ε**^a^	3.0	3.6	3.5	3.4	3.8	4.0
**λ_em_ [nm]**	713	712	719	685	683	662
***Φ_F_* [%]**	0.6	0.6	0.7	1.3	1.8	3.8
***Φ*_Δ_ [%]**^b^	30	29	31	NA	NA	NA

^a^L·mol^-1^·cm^-1^ × 10^4^. ^b^Determined by ^1^O_2_ phosphorescence emission in deuterated PBS. PTTP-DCns: Benzene-pyridothiadiazole-thienothiophene-pyridothiadiazole-benzene conjugated framework with quaternary ammonium-terminated n-carbon alkyl chains at both ends; PBS: phosphate-buffered saline.

Subsequently, the photosensitizing abilities of PTTP-DCns and PTTP-DCn@Ls were assessed by measuring the ^1^O_2_ generation under light irradiation. The ^1^O_2_ quantum yields (*Φ*_Δ_) of PTTP-DCns were first determined by directly measuring the ^1^O_2_ phosphorescence in deuterated PBS according to a previously reported method^[27]^, with commercial photosensitizer RB (*Φ*_Δ_ ≈ 76%) as the standard [Figure 1E, Supplementary Figures 5 and 6]. The *Φ*_Δ_ values of PTTP-DC4, PTTP-DC6, and PTTP-DC8 were calculated to be 30%, 29%, and 31%, respectively [Table 1]. Then, the ^1^O_2_ generation mediated by PTTP-DCns and PTTP-DCn@Ls over time was evaluated by the SOSG probe, which can be transformed into emissive endoperoxides with fluorescence peak around 525 nm after selective oxidization by ^1^O_2_. The SOSG fluorescence intensities at 525 nm in the presence of PTTP-DCns, PTTP-DCn@Ls, RB, and another commercial photosensitizer MB in PBS buffer were recorded over time under white light irradiation. As shown in Figure 1F, PTTP-DCn@Ls were able to produce ^1^O_2_, although the sensitizing ability was reduced compared to the corresponding PTTP-DCns.

### Interaction between MDR cells and PTTP-DCns

**Figure 2:**
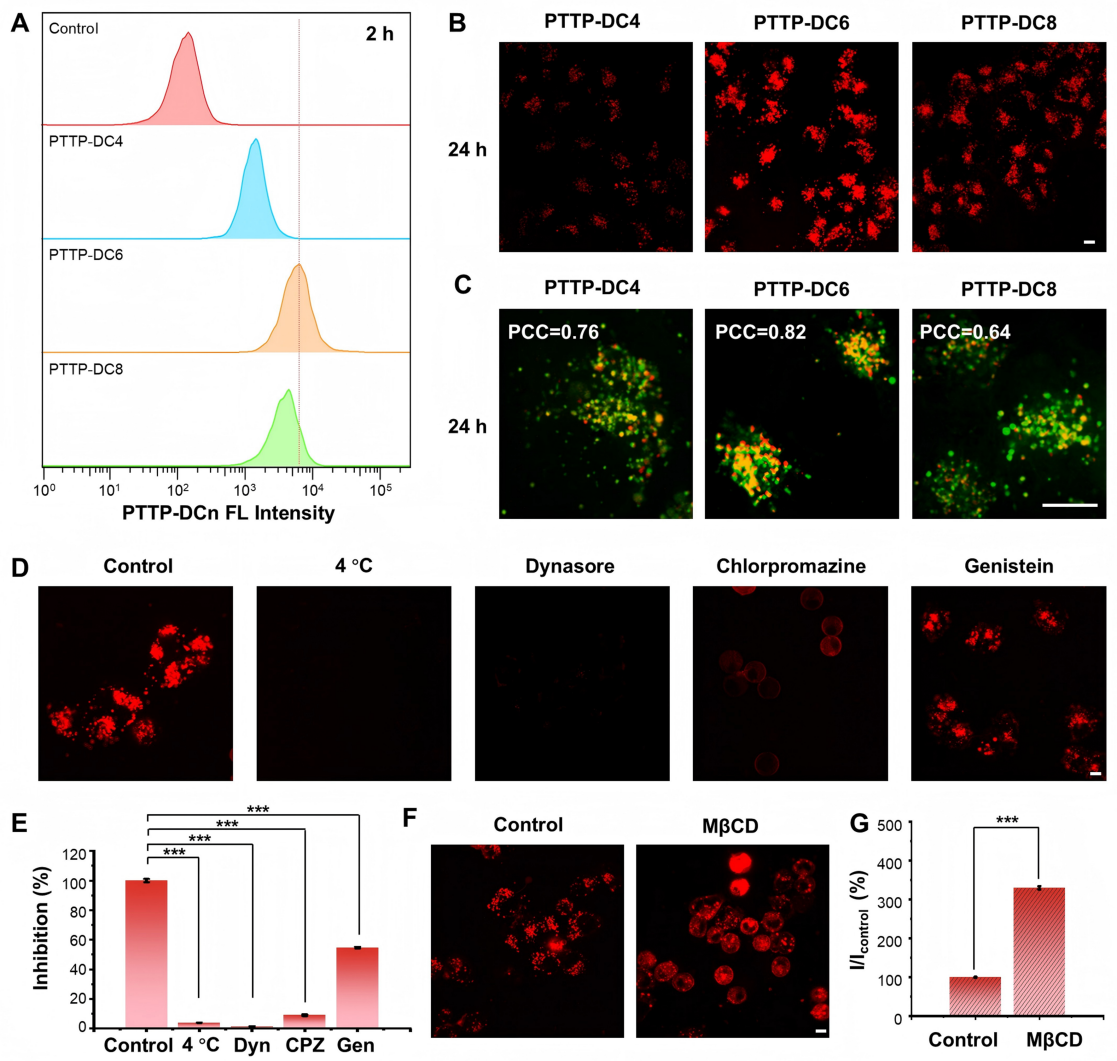
Cellular accumulation of PTTP-DCns. (A) Flow cytometric analysis of the cellular uptake of PTTP-DCns in MCF-7/ADR cells after a 2-hour incubation; (B) Confocal images of MCF-7/ADR cells stained with PTTP-DCns molecules after 24-hour incubation; (C) Representative merged images showing the co-localization of PTTP-DCns (red) and lysosome-specific dye LysoTracker Green (green) in MCF-7/ADR cells; (D and E) Confocal images and corresponding quantitative analysis (one-way ANOVA with Tukey’s test, ^***^P < 0.001) of MCF-7/ADR cells incubated with PTTP-DC6 (1 µM) in the presence of various endocytosis inhibition conditions; (F and G) Confocal images and corresponding quantitative analysis (Student’s *t*-test, ^***^P < 0.001) of MCF-7/ADR cells pre-treated with MβCD (10 mM) for 30 min and then incubated with PTTP-DC6 (10 µM) at 37 °C for 2 h. Scale bar: 10 μm. Data presented as mean ± SD. PTTP-DCns: Benzene-pyridothiadiazole-thienothiophene-pyridothiadiazole-benzene conjugated framework with quaternary ammonium-terminated n-carbon alkyl chains at both ends; MCF-7/ADR: Michigan Cancer Foundation-7/adriamycin-resistant; ANOVA: analysis of variance; PTTP-DC4/6/8: benzene-pyridothiadiazole-thienothiophene-pyridothiadiazole-benzene conjugated framework with quaternary ammonium-terminated C4/C6/C8 alkyl chains at both ends; PCC: Pearson’s correlation coefficient; MβCD: methyl-β-cyclodextrin; SD: standard deviation; FL: fluorescence; Dyn: dynasore; CPZ: chlorpromazine; Gen: genistein.

Next, the cellular uptake of PTTP-DCns was investigated using flow cytometry and CLSM, with DOX-resistant (MCF-7/ADR) breast cancer cells as models. As the results showed, PTTP-DC6 exhibited the highest cellular uptake and membrane assembly efficiency [Figure 2A and B, Supplementary Figures 7 and 8]. The subcellular localization of PTTP-DCns was further examined via colocalization studies due to evident endocytosis. As shown in Figure 2C and Supplementary Figure 9, significant fluorescence overlap was observed between PTTP-DCns and lysosome dye LysoTracker Green after 24-hour incubation in MCF-7/ADR cells. Importantly, PTTP-DC6 also demonstrated the highest pearson’s correlation coefficient (PCC of 0.82), confirming that its internalization occurred primarily within lysosomes.

To investigate the internalization pathway of PTTP-DC6, PTTP-DC6/cell assembly was evaluated under various endocytosis inhibition conditions^[33]^. As shown in Figure 2D and E, no fluorescence emission of PTTP-DC6 in cells was observed under 4 °C incubation, indicating an energy-dependent cellular uptake process of PTTP-DCns. Meanwhile, the uptake of PTTP-DC6 was almost completely inhibited by dynamin inhibitors, Dyn, and CPZ, suggesting clathrin-mediated endocytosis. It is interesting to find that slight fluorescence of PTTP-DC6 can be observed in the CPZ-treated group and specifically located on cell membranes. Considering the ability of CPZ to enhance membrane fluidity^[34]^, the impact of membrane fluidity on MICOE assembly was validated by pre-treating cells with methyl-β-cyclodextrin (MβCD), as MβCD can increase membrane fluidity by eliminating cholesterol. Remarkably enhanced interaction of PTTP-DC6 with cell membranes was observed on MβCD-treated cells, demonstrating our suspicion [Figure 2F and G]. Hence, the slightly reduced PTTP-DC6 uptake by caveolae inhibitor Gen is probably a result of Gen-induced reduction of membrane fluidity^[35]^.

**Figure 3:**
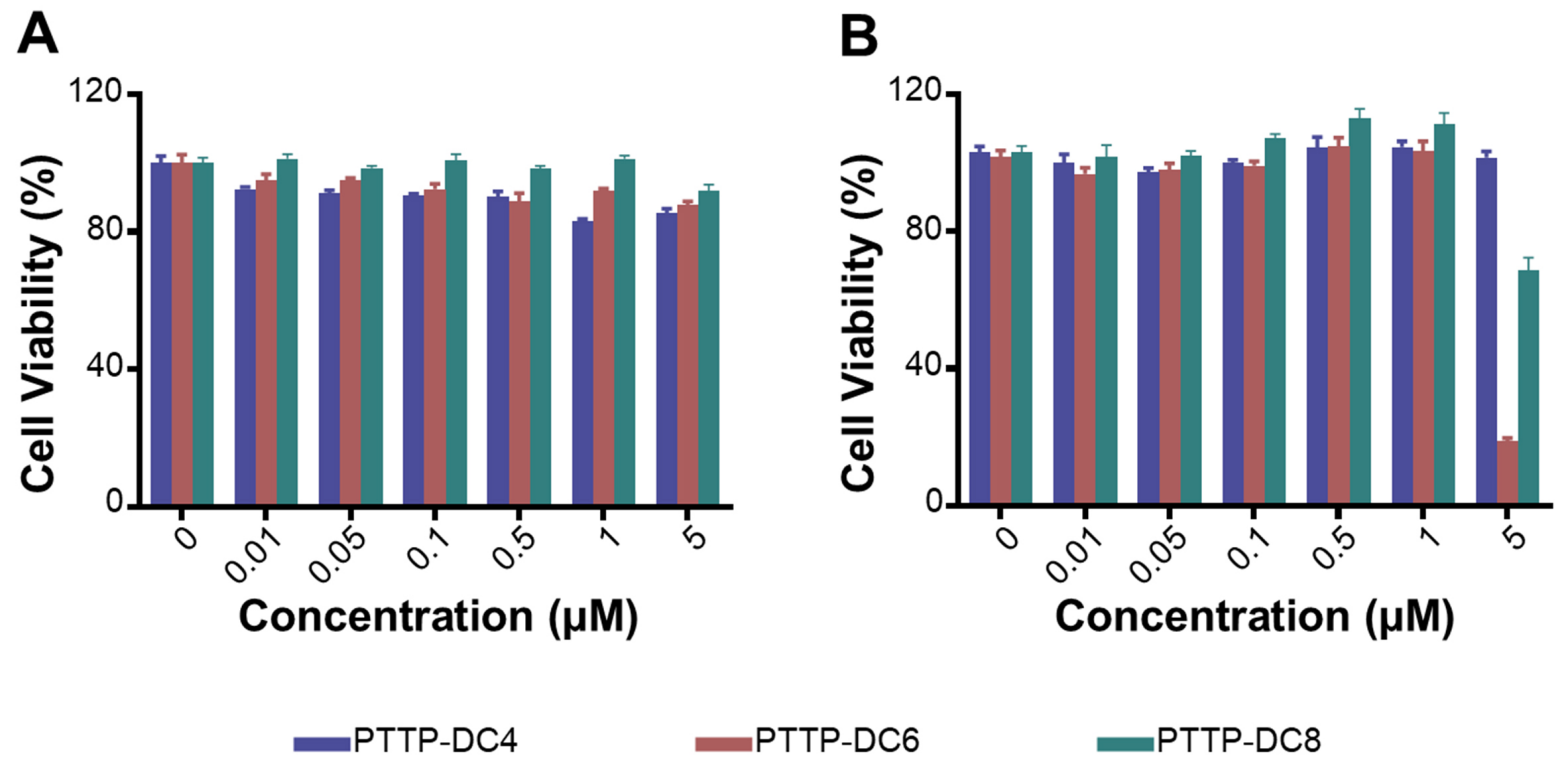
Biosafety assessment of PTTP-DCns *in vitro*. (A) Cell viabilities of MCF-7/ADR cells after being treated with different concentrations of PTTP-DCns for 48 h in the dark; (B) Cell viabilities of MCF-7/ADR cells pretreated with different concentrations of PTTP-DCns for 24 h, followed by the irradiation of a 525 nm LED light (0.2 mW·cm^-2^, 30 min) and incubated for another 24 h. Data presented as mean ± SD. PTTP-DCns: Benzene-pyridothiadiazole-thienothiophene-pyridothiadiazole-benzene conjugated framework with quaternary ammonium-terminated n-carbon alkyl chains at both ends; MCF-7/ADR: Michigan Cancer Foundation-7/adriamycin-resistant; LED: light emitting diode; SD: standard deviation; PTTP-DC4/6/8: benzene-pyridothiadiazole-thienothiophene-pyridothiadiazole-benzene conjugated framework with quaternary ammonium-terminated C4/C6/C8 alkyl chains at both ends.

Subsequently, the biosafety of PTTP-DCns towards cells was estimated via MTT assay. As shown in Figure 3A and Supplementary Figure 10, PTTP-DCns showed no significant dark toxicity to cancer cell line MCF-7/ADR and normal cell line HEK 293 (embryonic kidney cells) in the concentration range of 0 to 5 μM. Additionally, PTTP-DCns at concentrations below 1 μM barely affected cell viability under low-dose light exposure (525 nm LED, 0.2 mW·cm^-2^, 30 min) [Figure 3B]. Notably, at 5 μM, PTTP-DC6 demonstrates the strongest photocytotoxicity, which correlates with its superior cellular enrichment. Hence, 1 μM was chosen as the optimal concentration for the following study due to its good biosafety under both dark and mentioned light irradiation.

### Disruption of the lysosomal membrane by PTTP-DC6

**Figure 4:**
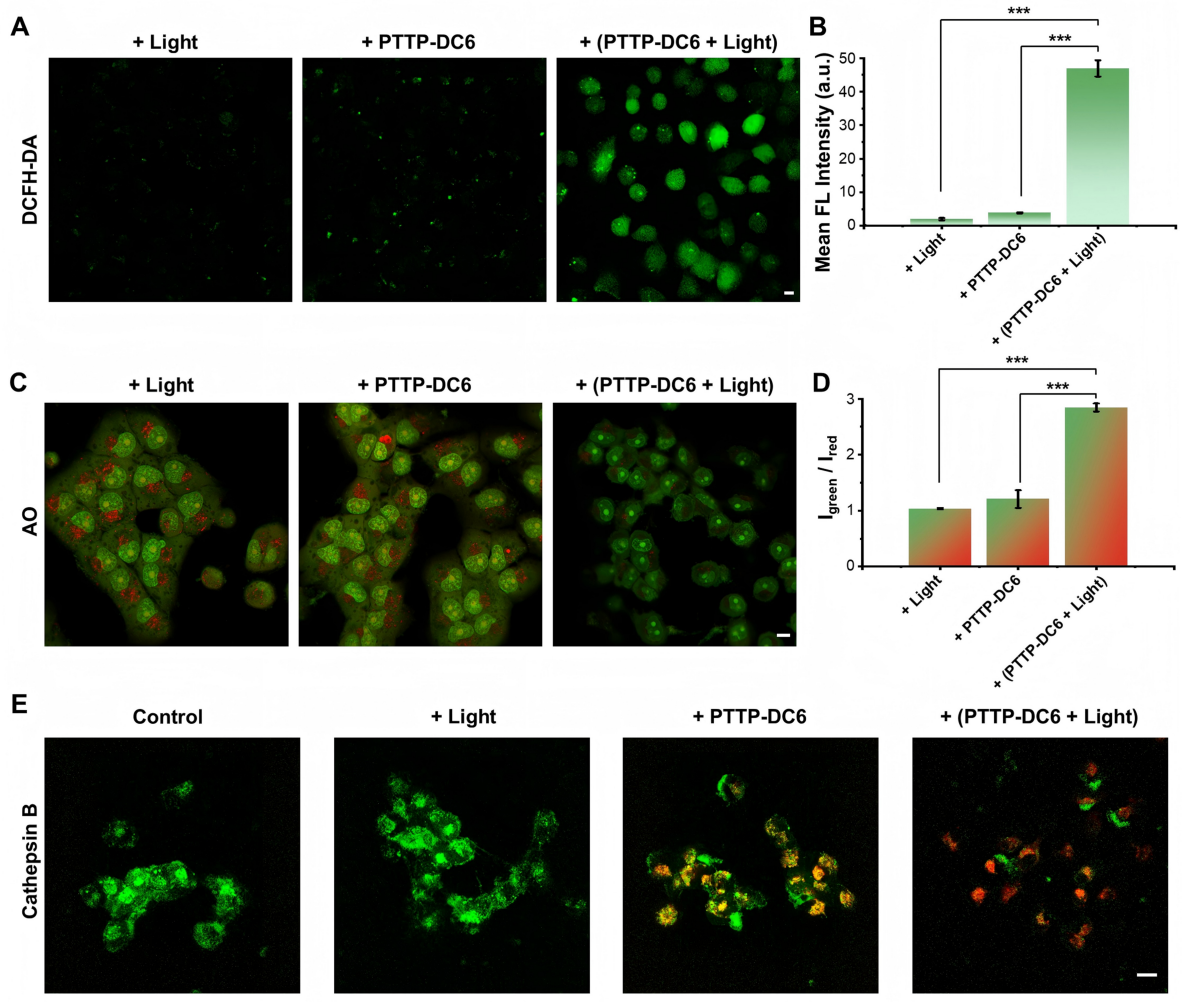
ROS generation and LMP induced by PTTP-DC6. (A and B) CLSM images and quantitative analysis (one-way ANOVA with Tukey’s test, ^***^P < 0.001) of ROS generation in MCF-7/ADR cells after PTTP-DC6 treatment and following irradiation. DCFH-DA was used as the ROS indicator; (C and D) CLSM images and quantitative analysis (one-way ANOVA with Tukey’s test, ^***^P < 0.001) of AO staining in MCF-7/ADR cells after various pretreatment. I_green_/I_red_: ratio of green-to-red mean fluorescence intensity in the cytoplasmic region; (E) CLSM images for assessment of cathepsin B activity in MCF-7/ADR cells after PTTP-DC6 treatment and following irradiation. Scale bar: 10 μm. Data presented as mean ± SD. ROS: Reactive oxygen species; LMP: lysosomal membrane permeabilization; PTTP-DC6: benzene-pyridothiadiazole-thienothiophene-pyridothiadiazole-benzene conjugated framework with quaternary ammonium-terminated C6 alkyl chains at both ends; CLSM: confocal laser scanning microscope; ANOVA: analysis of variance; MCF-7/ADR: Michigan Cancer Foundation-7/adriamycin-resistant; DCFH-DA: 2′,7′-dichlorodihydrofluorescein diacetate; AO: acridine orange; SD: standard deviation; FL: fluorescence.

Having confirmed the specific lysosomal localization and efficient enrichment of PTTP-DC6 within lysosomes, we next proceeded to investigate its mechanism for controlled membrane disruption. The photosensitizing ability of PTTP-DC6 after assembly with MDR cells was first investigated. DCFH-DA, a green fluorescence-emitting ROS indicator, was used to detect the intracellular ROS level via CLSM imaging. Upon ultra-low dose light exposure (525 nm, 0.2 mW·cm^-2^, 30 min), PTTP-DC6 induced an approximately 12-fold increase in DCF fluorescence intensity in MCF-7/ADR cells compared to the dark control, while the light-only group (+Light) showed no significant fluorescence [Figure 4A and B].

Next, we evaluated lysosomal integrity in PTTP-DC6-treated MCF-7/ADR cells using acridine orange (AO) staining, a method based on the environment-sensitive spectral shift of AO from red fluorescence in acidic lysosomes to green upon diffusion into the neutral cytosol. As shown in Figure 4C, cells in the light-only pretreated group exhibited strong red lysosomal fluorescence with diffuse green cytoplasmic signal. Quantitative analysis showed that PTTP-DC6 alone moderately increased the cytosolic green-to-red fluorescence ratio, which was further amplified approximately 2.4-fold upon light irradiation [Figure 4D], accompanied by a marked loss of red fluorescence. Since lysosomes are key to macromolecular degradation, membrane compromise can lead to leakage of hydrolytic enzymes such as cathepsin B into the cytosol^[36]^. To investigate whether the insertion and photoactivation of PTTP-DC6 trigger the release of lysosomal contents, we employed a Green Cathepsin B Assay Kit. As depicted in Figure 4E, obvious punctate green fluorescence, resulting from cleavage of the cathepsin B-specific substrate, was observed in both control and light-only groups. Cells pretreated with 1 μM PTTP-DC6 alone exhibited slightly attenuated and more dispersed green fluorescence, yet it still co-localized significantly with the red fluorescence of PTTP-DC6. Upon light irradiation (0.2 mW·cm^-2^, 30 min), the PTTP-DC6-pretreated cells showed a pronounced attenuation of the green signal, confirming extensive cathepsin B release resulting from ROS-induced LMP.

**Figure 5:**
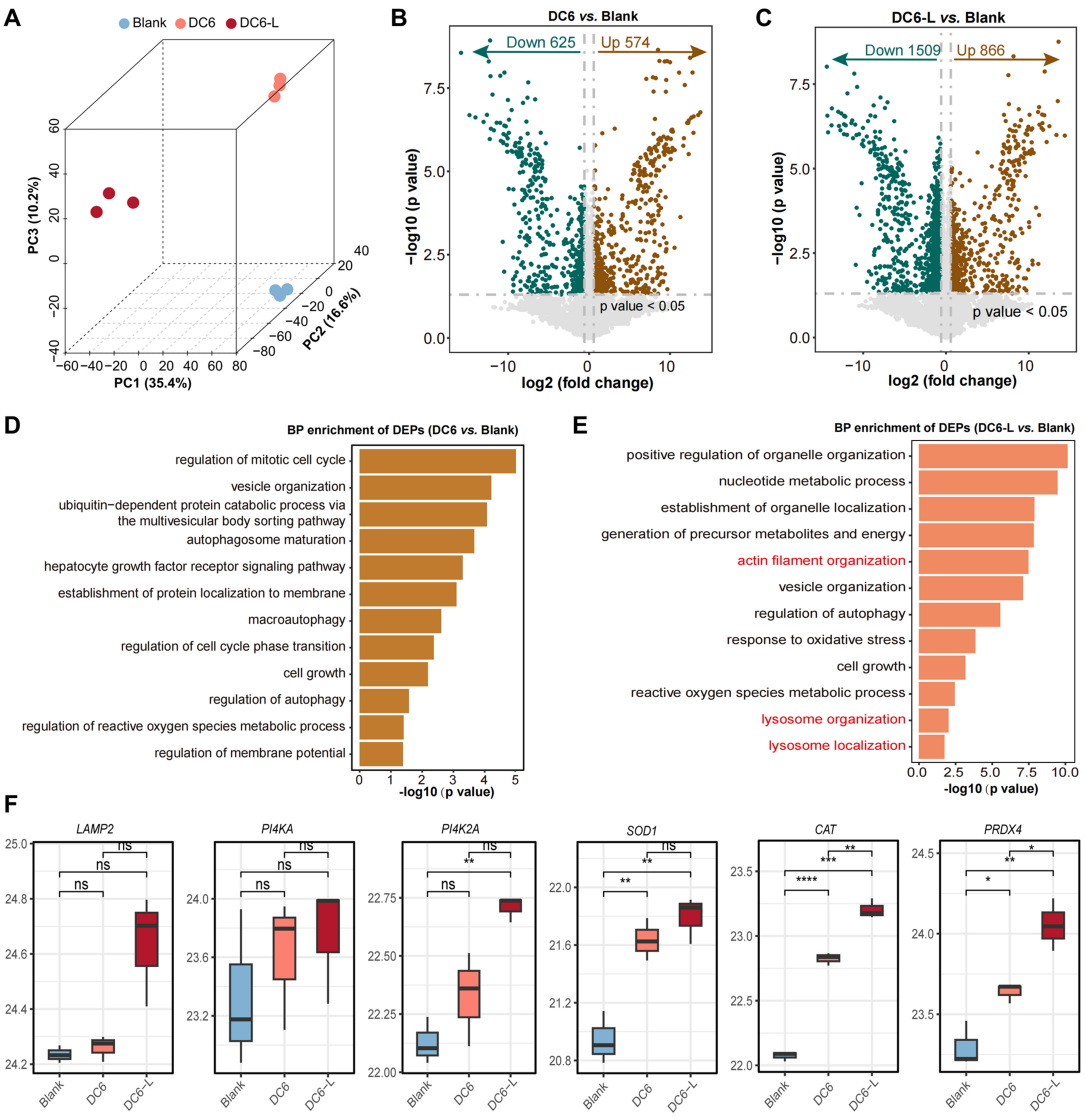
PTTP-DC6 triggers lysosomal impairment via a dual mode of action. (A) Three-dimensional PCA score plot of samples; (B and C) Volcano plots display DEPs in DC-6 *vs.* Blank and DC-6-L *vs.* Blank comparisons; (D) BP enrichment of DEPs in DC-6 *vs.* Blank; (E) BP enrichment of DEPs in DC-6-L *vs.* Blank; (F) Expression levels of lysosomal membrane proteins and ROS metabolism-related proteins (one-way ANOVA with Tukey’s test, ^*^P< 0.05; ^**^P < 0.01; ^***^P < 0.001; ^****^P < 0.0001. ns: no significance). PTTP-DC6: Benzene-pyridothiadiazole-thienothiophene-pyridothiadiazole-benzene conjugated framework with quaternary ammonium-terminated C6 alkyl chains at both ends; PCA: principal component analysis; DEPs: differentially expressed proteins; BP: biological process; ROS: reactive oxygen species; ANOVA: analysis of variance.

To elucidate the molecular mechanisms underlying the enhanced LMP induced by PTTP-DC6 under both dark and light, we performed label-free proteomic analysis^[37-39]^ in MCF-7/ADR cells. Principal component analysis (PCA) revealed distinct proteomic profiles among the blank, PTTP-DC6, and PTTP-DC6 plus light groups, with light exposure amplifying the proteomic alterations induced by PTTP-DC6 [Figure 5A-C]. Furthermore, biological process (BP) enrichment analysis highlighted that PTTP-DC6 treatment alone primarily affected pathways related to autophagy, membrane protein localization, and vesicle organization [Figure 5D]. In contrast, the DC6-L *vs.* Blank comparison showed marked enrichment in pathways linked to LMP and oxidative stress - including actin filament organization, lysosome organization, lysosome localization, ROS metabolic processes, and response to oxidative stress [Figure 5E]. Subsequently, we analyzed the expression of LMP- and ROS-related marker proteins. Interestingly, both LMP-related proteins (lysosome-associated membrane protein 2, LAMP2; phosphatidylinositol 4-kinase alpha, PI4KA; phosphatidylinositol 4-kinase type 2 alpha, PI4K2A) and ROS-related proteins (superoxide dismutase 1, SOD1; catalase, CAT; peroxiredoxin 4, PRDX4) showed a similar trend, with expression being upregulated by PTTP-DC6-only treatment and further enhanced under light exposure [Figure 5F].

### Recovering anticancer activity of DOX in MDR cancer cells and spheroids

**Figure 6:**
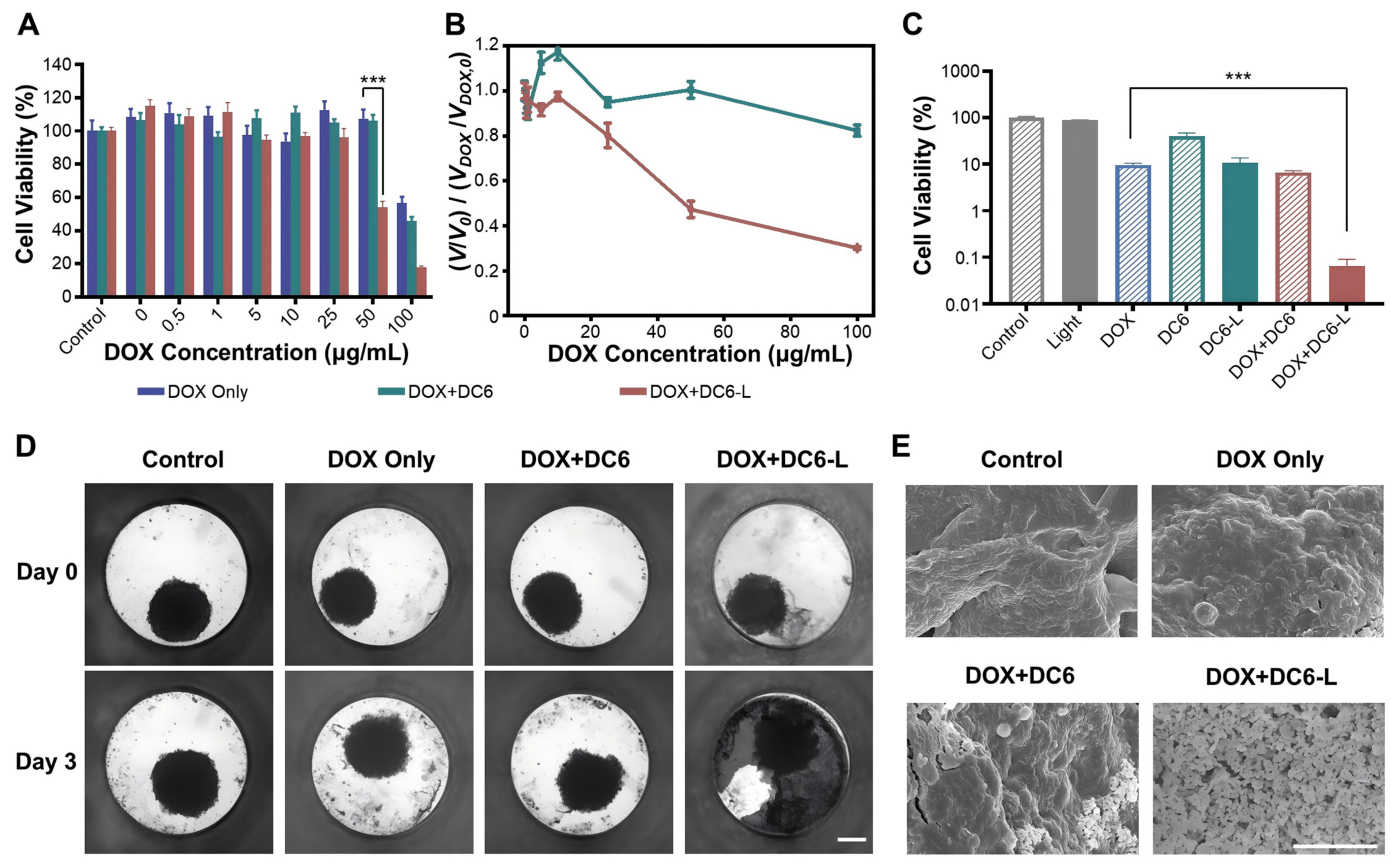
PTTP-DC6 enhances DOX efficacy in drug-resistant cells and spheroids. (A) Cytotoxicity of DOX to MCF-7/ADR cells after PTTP-DC6 (1 μM, 24 h) treatment under dark or light irradiation (525 nm, 0.2 mW·cm^-2^, 30 min) (one-way ANOVA with Tukey’s test, ^***^P < 0.001); (B) Relative viability rates of PTTP-D6-treated group under dark or light irradiation referring to viabilities of control group treated with DOX only. *V*: The cell viability of combinations, *V_DOX_*: the cell viability of DOX only, *V*_0_, *V_DOX_*_,0_: the cell viability in the 0 μM DOX group; (C) *In vitro* cytotoxicity of DOX (100 μg·mL^-1^) to MCF-7/ADR cell spheroids for 48 h with or without the pretreatment of PTTP-DC6 (5 μM, 24 h) and the followed irradiation of a 525 nm LED light (1 mW·cm^-2^, 30 min) (one-way ANOVA with Tukey’s test, ^***^P < 0.001); (D) Representative bright-field images of MCF-7/ADR cell spheroids with different treatments on day 0 and day 3. Scale bar: 200 µm; (E) Corresponding SEM images of MCF-7/ADR cell spheroids of various treatment conditions on day 3. Scale bar: 10 µm. Data presented as mean ± SD. PTTP-DC6: Benzene-pyridothiadiazole-thienothiophene-pyridothiadiazole-benzene conjugated framework with quaternary ammonium-terminated C6 alkyl chains at both ends; DOX: doxorubicin; MCF-7/ADR: Michigan Cancer Foundation-7/adriamycin-resistant; ANOVA: analysis of variance; LED: light emitting diode; SEM: scanning electron microscope; SD: standard deviation.

We first evaluate the ability of PTTP-DCns to reverse drug resistance in cancer cells using the MTT assay. The viabilities of MCF-7/ADR cells treated with DOX at various concentrations and 1 μM PTTP-DCns under dark or light irradiation were tested [Figure 6A, Supplementary Figure 11A and B]. With the viabilities of cells treated with DOX only in the dark as references, the corresponding ratios were calculated for quantitative analysis [Figure 6B, Supplementary Figure 11C and D]. PTTP-DC6 and PTTP-DC8 exhibited the ability to recover drug sensitivity of MCF-7/ADR cells under light, while PTTP-DC4 did not possibly due to weak association [Supplementary Figure 8]. However, only PTTP-DC6 decreased the cell viability under dark. As shown in Figure 6B, the introduction of PTTP-DC6 decreased the viability of cells treated with 100 μg·mL^-1^ DOX by 19% in the dark, and additional light processing further decreased the viability by 68%. The half-maximal inhibitory concentration (IC_50_) for DOX in PTTP-DC6 pre-treated MCF-7/ADR cells under light decreased to 48.6 μg·mL^-1^, with a resistance index (RI) decreasing from 39 to 19.

In addition, we compared the resensitization efficacy and cytotoxicity of PTTP-DC6 with CQ, a classical lysosomal disruptor [Supplementary Figure 12]. Although CQ showed a stronger resensitization effect than PTTP-DC6 treatment in the dark, the combination of PTTP-DC6 with light achieved the highest efficacy. Notably, CQ exhibited significant cytotoxicity at 1 μM. To further validate the mechanistic specificity of our approach, we established an icotinib-resistant HCC827 cell line (HCC827/IR), in which resistance is primarily mediated by the epidermal growth factor receptor (EGFR) T790M mutation. As shown in Supplementary Figure 13, pretreatment with PTTP-DC6 under either dark or light conditions did not re-sensitize these cells to icotinib.

Given the challenges in establishing reliable animal models of MCF-7/ADR tumors, we utilized three-dimensional (3D) cell spheroids to better mimic tumors *in vivo* and evaluate the drug sensitivity-restoring effect of PTTP-DC6. Compared with traditional two-dimensional (2D) monolayers, 3D tumor spheroids are better models to reflect drug efficacy *in vivo*, achieving more efficient and cost-effective preclinical screening of anticancer drugs^[40,41]^. The 3D cell spheroids were formed by 4 days’ culturing in Akura™ PLUS Hanging Drop Plate. 1 day after transferring to Akura™ 96 Spheroid Microplate, cell spheroids were treated with blank cell medium or 5 μM PTTP-DC6 for 24 h, following by light irradiation or dark incubation for 30 min. Notably, the fluorescence emitted by PTTP-DC6 and DOX in these spheroids significantly interfered with the detection of conventional fluorescence dyes such as calcein and propidium iodide (PI), hindering the accurate assessment of MCF-7/ADR cell survival using live-dead staining assays. Therefore, a viability assay using CellTiter-Glo® kit was applied to determine the survival rates after another 48-hour incubation with 100 μg·mL^-1^ DOX. Consistent with the trend in 2D monolayers [Figure 6A and B], PTTP-DC6-treated cell spheroids were more fragile to DOX compared to untreated cell spheroids under dark. Light irradiation could further decrease the cell viability by 2 orders to almost total death compared to cells spheroids treated with DOX or PTTP-DC6 only [Figure 6C]. The morphology of cell spheroids was further examined on a high-content imaging system and a scanning electron microscope (SEM). Notably, the loss of apparent microtissue morphology was not evident for all the groups, except the group treated with PTTP-DC6, DOX, and light irradiation, which exhibited obvious cellular debris dispersion [Figure 6D and Supplementary Figure 14]. The details of 3D cell spheroids were imaged using SEM. The cell spheroids treated with DOX or PTTP-DC6 only slightly increased the surface roughness as compared to the controls, while small portion of debris can be observed on the cell spheroid treated with both DOX and PTTP-DC6. Notably, the introduction of PTTP-DC6 together with light irradiation could violently trigger the cell lysis to small debris in the presence of DOX [Figure 6E and Supplementary Figure 15].

## DISCUSSION

In this study, we developed a series of MICOE-based photosensitizers (PTTP-DCns) with varying side chain lengths to overcome lysosomal sequestration-mediated MDR via a dual-mode mechanism. All PTTP-DCns display significantly increased zeta potentials, enhanced fluorescence and moderate photosensitizing abilities upon insertion into lipid bilayers. These findings indicate that PTTP-DCns in the membrane are capable of generating moderate ^1^O_2_ upon light excitation, enabling the light-regulation of membrane permeabilization as proposed in Scheme 1C.

A key finding of our work is the significant impact of side-chain length on cellular interaction and therapeutic efficacy. Studies on the interaction of PTTP-DCns with MCF-7/ADR cells reveal that PTTP-DC6 exhibits the highest lysosomal membrane assembly efficiency, which is likely attributable to its optimal side chain length (C6) that balances hydrophobic insertion and electrostatic interaction with the lipid bilayer. This structure-dependent behavior underscores the critical role of molecular design in the assembly of MICOEs with lipid membranes. Furthermore, the endocytosis mechanism studies reveal that the internalization of PTTP-DC6 is mainly mediated by clathrin-related endocytosis and affected obviously by membrane fluidity, providing insights into the uptake pathway of such amphiphilic molecules.

The core mechanism by which PTTP-DC6 overcomes lysosomal sequestration-mediated MDR involves the disruption of lysosomal membrane through a dual-mode action. We observed that PTTP-DC6 alone could induce mild LMP in MCF-7/ADR cells, while light irradiation significantly amplified this effect via ROS generation. These results demonstrate that even a low dose of PTTP-DC6 under mild conditions (darkness or weak light) can induce LMP in MDR cells, highlighting its potential to restore the efficacy of DOX by overcoming lysosomal drug sequestration. The proteomic analysis further confirmed this mechanism. The coordinated upregulation of antioxidant enzymes (SOD1, CAT, PRDX4) suggests that PTTP-DC6 treatment induces a state of oxidative stress, which is a well-known upstream inducer of LMP^[42]^. Moreover, the increased expression of LMP-related proteins (LAMP2 and PI4K2A) points to a cellular compensatory/reparative response to membrane damage^[43,44]^. These proteomic findings indicate that treatment with PTTP-DC6, whether in darkness or under low-dose light, can trigger proteomic alterations associated with enhanced ROS metabolism and lysosomal dysfunction in MDR cancer cells, providing mechanistic support for its dual-mode action of PTTP-DC6. Specifically, we propose that the mechanism by which PTTP-DC6 overcomes cancer drug resistance involves: (i) its physical association with lysosomal membranes increases LMP under both dark and light conditions; (ii) light exposure further enhances LMP via lipid peroxidation initiated by *in situ* ROS generation.

Subsequently, the ability of PTTP-DC6 to reverse lysosomal sequestration-mediated drug resistance was validated through drug resensitization assays. The IC_50_ for DOX in PTTP-DC6 pre-treated MCF-7/ADR cells under light decreased significantly, with the RI dropping from 39 to 19. These results reveal a clear side-chain length–dependent structure–activity relationship. Only the C6 chain achieves the optimal balance between lysosomal integration and membrane perturbation, enabling dual-mode drug resensitization. In contrast, the C4 chain is too short for stable anchoring, and the C8 chain is too long to efficiently disrupt the membrane. When compared to CQ, a classical lysosomal disruptor, PTTP-DC6 combined with light achieved superior efficacy while maintaining excellent biosafety, as CQ exhibited significant cytotoxicity at effective concentrations. Additionally, the failure of PTTP-DC6 to re-sensitize icotinib-resistant cells (where resistance is not lysosome-dependent) confirms the specificity of our strategy towards lysosomal drug sequestration.

Finally, the efficacy of PTTP-DC6 was confirmed in MDR cancer spheroids, a model that mimics the physiological environment of solid tumors. The cell viability and morphology results illustrate that PTTP-DC6 can significantly improve the anticancer efficacy of DOX in a 3D *in vitro* model of MDR cells, further validating the dual-mode mechanism in a more physiologically relevant context. Nevertheless, the absence of *in vivo* validation remains a key limitation of this study. The translational potential of PTTP-DC6, therefore, necessitates future evaluation in clinically relevant patient-derived xenograft (PDX) models of resistant tumors. To enable tumor-targeted delivery *in vivo*, future efforts could leverage hybrid cell membrane-coated nanoparticles^[45]^ as a co-delivery system, with DOX encapsulated in the core and PTTP-DC6 anchored in the membrane, to achieve synergistic lysosomal disruption. Additionally, clinical translation of this photodynamic approach would require strategies to overcome the limited tissue penetration of the green light used here, which could be achieved by developing next-generation MICOE photosensitizers activatable by longer-wavelength or two-photon excitation.

In summary, this study presents a rational design of side-chain-engineered MICOEs that effectively reverse MDR by regulating lysosomal membrane functions. The dual-mode action of PTTP-DC6, which combines physical membrane intercalation in the dark with photochemical ROS generation under light, offers a promising strategy to enhance chemotherapy outcomes with high specificity and minimal toxicity. Overall, this work demonstrates the great potential of MICOEs as a versatile molecular platform for cancer therapy. Building on their unique topological structure and optical property, they establish a novel insight and strategy for overcoming drug resistance by synergistically regulating subcellular organelle membranes.

## DECLARATIONS

### Availability of data and materials

All datasets and materials generated in this study can be provided by the corresponding author (Bing Wang) upon reasonable request.

### AI and AI-assisted tools statement

During the preparation of this manuscript, the AI tool DeepSeek (version V3.2, released 2025-12-01) was used solely for language editing. The tool did not influence the study design, data collection, analysis, interpretation, or the scientific content of the work. All authors take full responsibility for the accuracy, integrity, and final content of the manuscript.

### Copyright

© The Author(s) 2026.
