# Supplementary material for: Conjugated oligoelectrolytes overcome cancer drug resistance by dual-mode lysosomal membrane disruption
Source: Cancer Drug Resist. 2026 Mar 20;9:8. doi: 10.20517/cdr.2025.196 (PMC13103252; doi:10.20517/cdr.2025.196)
Supplement: Supplementary file 1 [file cdr-9-8-SupplementaryMaterials.pdf]

Supplementary Materials

Conjugated oligoelectrolytes overcome cancer drug resistance by dual-mode lysosomal membrane disruption

Lingna Wang[1,2,3], Yufei Mao[2,4], Yu Dong[2,5], Manqi Tan[2,4], Xingyu Wang[2,3], Zhaobo Liu[2,4], Chenyao Nie[4], Shu Xing[2,3], Meng Li[2,3], Haitao Yuan[6], Bing Wang[2,3]

[1]Ningbo Institute for Drug Control, Ningbo 315048, Zhejiang, China.
[2]Laboratory of Advanced Theranostic Materials and Technology, Ningbo Institute of Materials Technology and Engineering, Chinese Academy of Sciences, Ningbo 315201, Zhejiang, China.
[3]Ningbo Cixi Institute of Biomedical Engineering, Ningbo 315300, Zhejiang, China.
[4]Cixi Biomedical Research Institute, Wenzhou Medical University, Wenzhou 315302, Zhejiang, China.
[5]College of Pharmaceutical Science, Zhejiang University of Technology, Hangzhou 310014, Zhejiang, China.
[6]Center for Drug Research and Development, Guangdong Provincial Key Laboratory for Research and Evaluation of Pharmaceutical Preparations, Guangdong Pharmaceutical University, Guangzhou 510006, Guangdong, China.

Correspondence to: Prof. Meng Li, Prof. Bing Wang, Laboratory of Advanced Theranostic Materials and Technology, Ningbo Institute of Materials Technology and Engineering, Chinese Academy of Sciences, Ningbo 315201, Zhejiang, China. E-mail: limeng@nimte.ac.cn; wangbing@nimte.ac.cn; Prof. Haitao Yuan, Center for Drug Research and Development, Guangdong Provincial Key Laboratory for Research and Evaluation of Pharmaceutical Preparations, Guangdong Pharmaceutical University, Guangzhou 510006, Guangdong, China. E-mail: yht193525@163.com

1. Materials and Methods

1.1 Materials

Unless stated otherwise, all analytical grade chemicals and solvents in this paper were purchased from commercial vendors and used without further purification. 1,2-distearoyl-sn-glycero-3-phosphocholine (DSPC), methylene blue (MB), rose bengal (RB), chlorpromazine (CPZ), genistein (GEN), dynasore (DYN), 2',7'-dichlorodihydrofluorescein diacetate (DCFH-DA), icotinib, and 3-(4,5-dimethylthiazol-2-yl)-2,5-diphenyltetrazolium bromide (MTT) were obtained from Aladdin (Shanghai, China). Singlet Oxygen Sensor Green (SOSG) was obtained from Meilunbio (China). Lyso-Tracker Green DND-26 was obtained from Invitrogen (USA). Acridine Orange hydrochloride hydrate (AO) was obtained from Sigma-Aldrich (USA). Green Cathepsin B Assay Kit was obtained from ImmunoChemistry Tech (USA). Doxorubicin (DOX) was obtained from Solarbio (Beijing, China). Penicillin-Streptomycin (P/S) Solution and fetal bovine serum (FBS) were purchased from TransGen Biotech (Beijing, China) and PAN-Biotech (UK), respectively. RPMI 1640 medium and high-glucose Dulbecco's Modified Eagle Medium (DMEM) were purchased from Gibco (USA). Phosphate buffered saline (PBS) was obtained from Corning (USA). Akura™ 96 Spheroid Microplate assay plates and CellTiter-Glo® 3D Reagent were obtained from InSphero (Swiss) and Promega (Beijing, China), respectively.

1.2 Apparatus and characterization

^{1}H NMR and ^{13}C NMR spectra were recorded on Bruker AVANCE NEO 400 and Bruker AVANCE NEO 600. HRMS spectra were recorded with a Time of Flight Mass Spectrometer (AB Sciex TripleTOF 4600). Absorption and fluorescence spectra were measured on an Agilent Cary 5000 UV-Vis-NIR spectrophotometer and Perkin-Elmer LS-55 fluorescence spectrophotometer, respectively. Dynamic light scattering and zeta potential measurements were performed on a nanoparticle size analyzer (Anton Paar GmbH Litesizer 500). White light illumination was performed using a fiber-optic xenon light source (Microenerg CME-303F) and the light density was determined using a TES-132 power meter. A 525 nm light irradiation was performed using a 525 nm LED cold light source (PerfectLight PLS-LED 100C) and a TES-132 power meter was used to determine the output light density. Cell viability was measured using a microplate reader (Tecan SPARK®). CLSM imagings were performed on a Leika TCS SP8 laser

scanning confocal microscope and an Andor Dragonfly 202 high-speed confocal microscope system. Flow cytometry was analyzed on a BD LSR Fortessa Flow Cytometer. 3D microtissues imaging was performed on an Operetta® High Content Imaging System (Perkin Elemer). Scanning electron microscopy (SEM) images were acquired using a FE-SEM microscope (Regulus 8230).

1.3 Synthesis

General procedure for the synthesis of compound 2-n:

1-Bromo-3,5-dihydroxybenzene (5.3 mmol) and K_2CO_3 (21.2 mmol) were dissolved in 15 mL of acetone and degassed with N_2 for 30 min. To the solution, the corresponding alkyl dibromide (26.5 mmol) was added, heated up to reflux, and stirred for 24 h. The mixture was filtered and washed with CH_2Cl_2. The filtrate was collected and the solvent was removed by rotary evaporation. The residue was purified by silica gel column chromatography using ethyl acetate/petroleum ether (v/v, 1/19) as eluent. The chemical structures of compounds **2-n** were confirmed by NMR and MS data.

Compound **2-4** was obtained as white solid in 73% yield: ^{1}H NMR (400 MHz, $CDCl_3$, δ): 6.64 (d, *J*= 2.2 Hz, 2H), 6.35 (t, *J*= 2.2 Hz, 1H), 3.95 (t, *J*= 6.0 Hz, 4H), 3.48 (t, *J*= 6.6 Hz, 4H), 2.08-2.01 (m, 4H), 1.96-1.89 (m, 4H). ^{13}C NMR (101 MHz, $CDCl_3$, δ): 160.6, 123.1, 110.6, 100.8, 67.3, 33.4, 29.5, 27.9. HR-MS (LC-Q-TOF): m/z $[M+H]^+$ calculated for $C_{14}H_{20}Br_3O_2^+$, 458.8988; found, 458.8974.

Compound **2-6** was obtained as white solid in 78% yield: ^{1}H NMR (600 MHz, $CDCl_3$, δ): 6.64 (s, 2H), 6.36 (s, 1H), 3.91 (t, *J*= 6.3 Hz, 4H), 3.42 (t, *J*= 6.8 Hz, 4H), 1.91-1.87 (m, 4H), 1.80-1.75 (m, 4H), 1.51-1.48 (m, 8H). ^{13}C NMR (101 MHz, $CDCl_3$, δ): 160.8, 123.0, 110.4, 100.7, 68.1, 33.9, 32.8, 29.1, 28.0, 25.4. HR-MS (LC-Q-TOF): m/z $[M+H]^+$ calculated for $C_{18}H_{28}Br_3O_2^+$, 514.9614; found, 514.9619.

Compound **2-8** was obtained as white solid in 70% yield: ^{1}H NMR (400 MHz, $CDCl_3$, δ): 6.63 (d, *J*= 2.2 Hz, 2H), 6.36 (t, *J*= 2.2 Hz, 1H), 3.90 (t, *J*= 6.5 Hz, 4H), 3.41 (t, *J*= 6.8 Hz, 4H), 1.90-1.82 (m, 4H), 1.78-1.71 (m, 4H), 1.48-1.41 (m, 8H), 1.36-1.34 (m, 8H). ^{13}C NMR (101 MHz, $CDCl_3$, δ): 160.9, 123.0, 110.4, 100.8, 68.3, 34.1, 32.9, 29.3, 29.2, 28.8, 28.2, 26.0. HR-MS (LC-Q-TOF): m/z $[M+H]^+$ calculated for $C_{22}H_{36}Br_3O_2^+$, 571.0240; found, 571.0240.

General procedure for the synthesis of compound 4-n: Under N_2 atmosphere, compound **2-n** (2.63 mmol) was dissolved in anhydrous THF (40 mL). After cooling down to -78 °C for 15 min in a dry ice/acetone bath, n-butyllithium (5.26 mmol, 2.4 M

in hexane) was added dropwise and the solution was kept at -78 °C for 1 h. Then tributyltin chloride (3.16 mmol) was added dropwise. After stirring for another 1 h, the reaction mixture was slowly warmed to room temperature and stirred overnight. Then saturated NH_4Cl aqueous solution was added to quench the reaction. The mixture was extracted with CH_2Cl_2, and the organic phase was collected and dried with anhydrous $MgSO_4$. The solvent was removed in vacuo to afford crude compound **3-n**, which was used directly in the next step without further purification. To 10 mL of anhydrous toluene, 4,7-dibromo-[1,2,5]thiadiazolo[3,4-c]pyridine (0.17 mmol), tris(dibenzylideneacetone) dipalladium (0) ($Pd_2(dba)_3$) (0.004 mmol), tri(*o*-tolyl) phosphine (P(*o*-tol)$_3$) (0.032 mmol) and compound **3-n** (0.13 mmol) were added in glove box. Then the solution was stirred for 24 h at 110 °C. After cooling down to room temperature, the mixture was poured into water and extracted with CH_2Cl_2. After dried with $MgSO_4$, the organic solvent was removed under vacuum, and the residue was purified by silica gel column chromatography using ethyl acetate/petroleum ether (v/v, 1/19) as eluent. The chemical structures of compounds **4-n** were confirmed by NMR and MS data.

Compound **4-4** was obtained as yellow solid in 34% yield: ^{1}H NMR (400 MHz, $CDCl_3$, δ): 8.81 (s, 1H), 7.77 (d, *J*= 2.3 Hz, 2H), 6.64 (t, *J*= 2.3 Hz, 1H), 4.11 (t, *J*= 6.0 Hz, 4H), 3.52 (t, *J*= 6.6 Hz, 4H), 2.15-2.08 (m, 4H), 2.03-1.97 (m, 4H). ^{13}C NMR (151 MHz, $CDCl_3$, δ): 160.4, 156.9, 152.3, 149.5, 145.7, 137.9, 110.2, 108.6, 104.5, 67.3, 33.6, 29.6, 28.0. HR-MS (LC-Q-TOF): m/z $[M+H]^+$ calculated for $C_{19}H_{21}Br_3N_3O_2S^+$, 593.8879; found, 593.8868.

Compound **4-6** was obtained as yellow solid in 12% yield: ^{1}H NMR (400 MHz, $CDCl_3$, δ): 8.80 (s, 1H), 7.75 (d, *J*= 2.2 Hz, 2H), 6.65 (t, *J*= 2.2 Hz, 1H), 4.07 (t, *J*= 6.4 Hz, 4H), 3.44 (t, *J*= 6.8 Hz, 4H), 1.93-1.83 (m, 8H), 1.55-1.52 (m, 8H). ^{13}C NMR (151 MHz, $CDCl_3$, δ): 160.5, 156.8, 152.5, 149.6, 145.7, 137.8, 110.1, 108.6, 104.5, 68.2, 33.9, 32.8, 29.2, 28.1, 25.5. HR-MS (LC-Q-TOF): m/z $[M+H]^+$ calculated for $C_{23}H_{29}Br_3N_3O_2S^+$, 649.9505; found, 649.9500.

Compound **4-8** was obtained as yellow solid in 39% yield: ^{1}H NMR (400 MHz, $CDCl_3$, δ): 8.80 (s, 1H), 7.74 (d, *J*= 2.2 Hz, 2H), 6.65 (t, *J*= 2.2 Hz, 1H), 4.06 (t, *J*= 6.4 Hz, 4H), 3.41 (t, *J*= 6.8 Hz, 4H), 1.90-1.79 (m, 8H), 1.52-1.42 (m, 8H), 1.39-1.36 (m, 8H). ^{13}C NMR (151 MHz, $CDCl_3$, δ): 160.6, 156.8, 152.6, 149.6, 145.7, 137.8, 110.0, 108.5, 104.5, 68.3, 34.1, 32.9, 29.4, 29.3, 28.8, 28.2, 26.1. HR-MS (LC-Q-TOF): m/z $[M+H]^+$

calculated for $C_{27}H_{37}Br_3N_3O_2S^+$, 706.0131; found, 706.0148.

General procedure for the synthesis of compound 5-n: To 10 mL of anhydrous toluene, compound **4-n** (0.14 mmol), 2,5-bis(trimethylstannyl)thieno[3,2-b]thiophene (0.056 mmol), tris(dibenzylideneacetone) dipalladium (0) ($Pd_2(dba)_3$) (0.006 mmol), and tri(*o*-tolyl) phosphine (P(*o*-tol)3) (0.048 mmol) were added in glove box. Then the solution was stirred for 24 h at 110 °C. After cooling down to room temperature, the mixture was poured into water and extracted with CH_2Cl_2. After dried with $MgSO_4$, the organic solvent was removed under vacuum, and the residue was purified by silica gel column chromatography using ethyl acetate/CH_2Cl_2 (v/v, 1/99) as eluent. The chemical structures of compounds **5-n** were confirmed by MS data. Due to the low solubility, NMR data were obtained only for compound **5-8**.

Compound **5-4** was obtained as fuchsia solid in 32% yield: HR-MS (LC-Q-TOF): m/z $[M+H]^+$ calculated for $C_{44}H_{43}Br_4N_6O_4S_4^+$, 1166.8916; found, 1166.8953.

Compound **5-6** was obtained as fuchsia solid in 54% yield: HR-MS (LC-Q-TOF): m/z $[M+H]^+$ calculated for $C_{52}H_{59}Br_4N_6O_4S_4^+$, 1279.0168; found, 1279.0257.

Compound **5-8** was obtained as red solid in 68% yield: ^1H NMR (400 MHz, $CDCl_3$, δ): 9.02 (s, 2H), 8.54 (s, 2H), 7.86 (s, 4H), 6.65 (s, 2H), 4.09 (t, *J*= 6.5 Hz, 8H), 3.42 (t, *J*= 6.6 Hz, 8H), 1.89-1.83 (m, 16H), 1.52-1.40 (m, 32H). HR-MS (LC-Q-TOF): m/z $[M+H]^+$ calculated for $C_{60}H_{75}Br_4N_6O_4S_4^+$, 1391.1420; found, 1391.1535.

General procedure for the synthesis of PTTP-DCns: Compound **5-n** (0.05 mmol) was dissolved in 5 mL of anhydrous THF and a large excess of trimethylamine solution in THF (2 M, ~1 mL) was added. Then the solution was stirred for 24 h at 40 °C in the dark. After cooling down to room temperature, the solvent was removed via bubbling with N_2. The crude was dissolved in degassed methanol and a large excess of trimethylamine solution in methanol (2 M, ~1 mL) was added and stirred for another 24 h at 40 °C in the dark. The solvent was removed by N_2 blowing, then the product was dissolved in deionized water, filtered by a 0.45 μm syringe filter and freeze-dried. The chemical structures of compounds **PTTP-DCns** were confirmed by NMR and MS data.

Compound **PTTP-DC4** was obtained as fuchsia solid in 83% yield: ^1H NMR (500 MHz, DMSO-d6, 373 K, δ): 9.16 (s, 2H), 8.67 (s, 2H), 7.91 (d, *J*= 2.3 Hz, 4H), 6.82 (t, *J*= 2.3 Hz, 2H), 4.22 (t, *J*= 6.2 Hz, 8H), 3.50-3.47 (m, 8H), 3.15 (s, 36H), 2.01-1.95 (m, 8H), 1.92-1.86 (m, 8H). ^{13}C NMR (126 MHz, DMSO-d6, 373 K, δ): 159.4, 154.0, 149.9, 148.5, 141.0, 139.8, 138.7, 137.8, 120.6, 120.4, 108.5, 103.9, 67.0, 65.2, 52.2,

25.4, 18.9. HR-MS (ESI): m/z $[M-4Br]^{4+}$ calculated for $C_{56}H_{78}N_{10}O_4S_{44}^+$, 270.6267; found, 270.6266.

Compound **PTTP-DC6** was obtained as fuchsia solid in 92% yield: ^1H NMR (600 MHz, DMSO-d6, δ): 9.19 (s, 2H), 8.69 (s, 2H), 7.84 (s, 4H), 6.71 (s, 2H), 4.09 (t, *J*= 6.0 Hz, 8H), 3.33-3.31 (m, 8H), 3.07 (s, 36H), 1.84-1.79 (m, 8H), 1.76-1.71 (m, 8H), 1.56-1.51 (m, 8H), 1.41-1.37 (m, 8H). ^{13}C NMR (151 MHz, DMSO-d6, δ): 159.9, 154.3, 149.9, 148.8, 141.5, 140.1, 139.2, 138.0, 120.9, 120.7, 108.1, 103.2, 67.6, 65.3, 52.2, 28.5, 25.6, 25.2, 22.1. HR-MS (ESI): m/z $[M-4Br]^{4+}$ calculated for $C_{64}H_{94}N_{10}O_4S_{44}^+$, 298.6580; found, 298.6579.

Compound **PTTP-DC8** was obtained as fuchsia solid in 90% yield: ^1H NMR (600 MHz, DMSO-d6, δ): 9.22 (s, 2H), 8.72 (s, 2H), 7.84 (s, 4H), 6.72 (s, 2H), 4.09 (t, *J*= 6.2 Hz, 8H), 3.29-3.26 (m, 8H), 3.04 (s, 36H), 1.81-1.77 (m, 8H), 1.72-1.66 (m, 8H), 1.48-1.47 (m, 8H), 1.39 (m, 16H), 1.33-1.29 (m, 8H). ^{13}C NMR (151 MHz, DMSO-d6, δ): 159.9, 154.3, 150.0, 148.9, 141.5, 140.2, 139.2, 138.0, 121.0, 120.7, 108.1, 103.3, 67.8, 65.3, 52.2, 28.7, 28.6, 28.5, 25.8, 25.5, 22.0. HR-MS (LC-Q-TOF): m/z $[M-4Br]^{4+}$ calculated for $C_{72}H_{110}N_{10}O_4S_{44}^+$, 326.6893; found, 326.6892.

2. Supplementary Figures

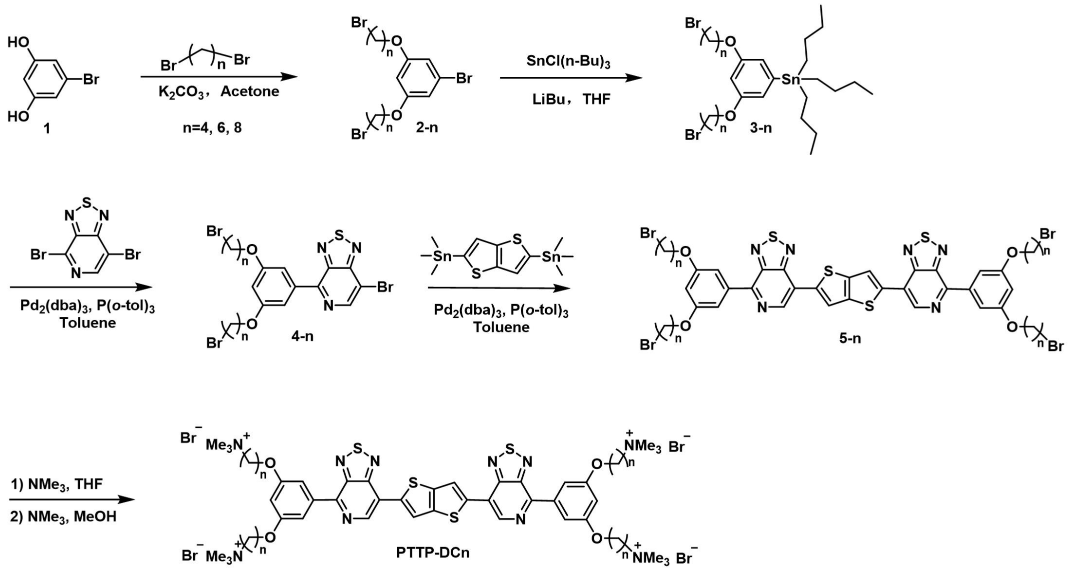

Supplementary Scheme 1. Synthetic route of **PTTP-DCns**.

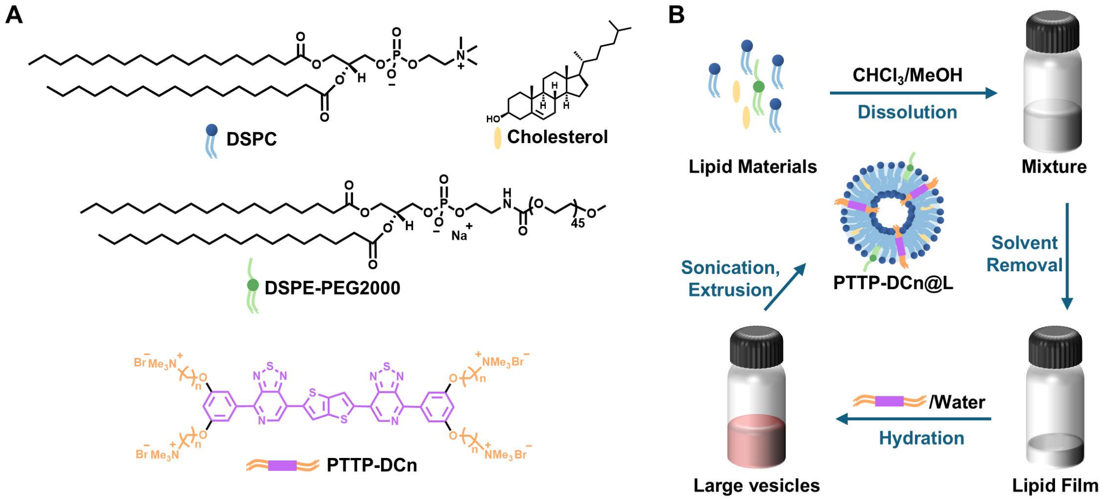

Supplementary Scheme 2. Schematic illustration for the preparation of **PTTP-DCn@Ls**. (A) Chemical structures of the components of PTTP-DCn@Ls; (B) Schematic illustration for the preparation of PTTP-DCn@Ls using the film hydration method.

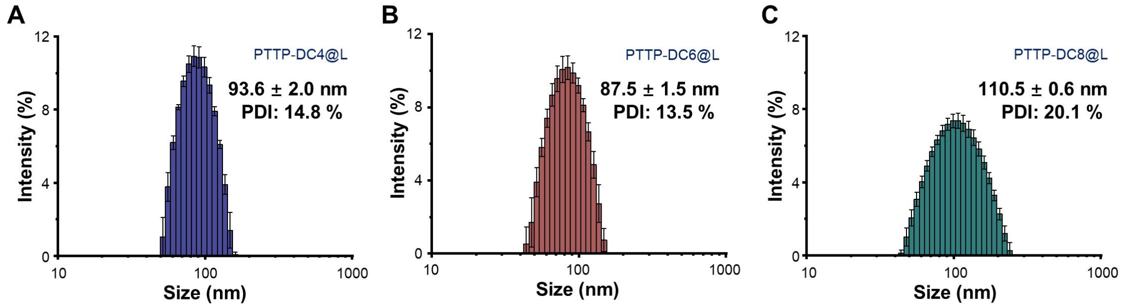

Supplementary Figure 1. DLS characterization of (A) PTTP-DC4@L, (B) PTTP-DC6@L and (C) PTTP-DC8@L in water. [PTTP-DCns] = 10 μM. Data presented as mean ± SD.

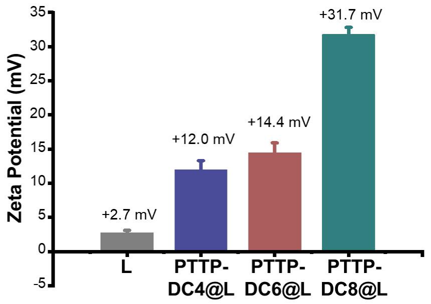

Supplementary Figure 2. Zeta potential of blank liposome and **PTTP-DCn@Ls** in water. [**PTTP-DCns**] = 10 μM. Data presented as mean ± SD.

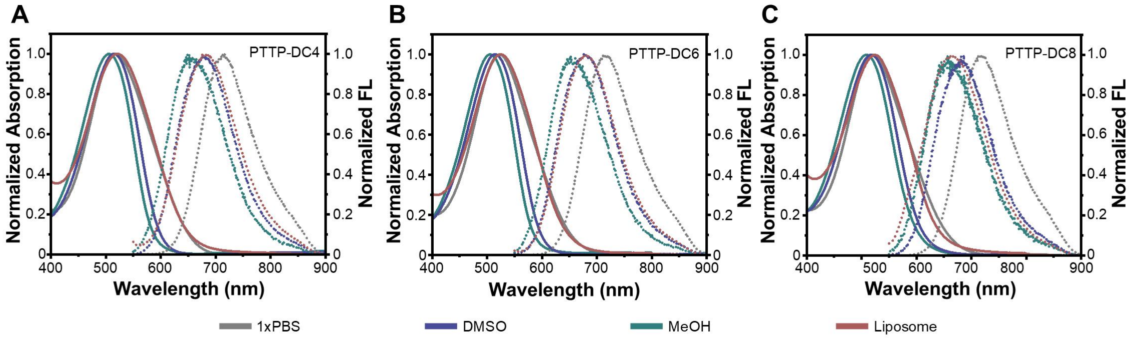

Supplementary Figure 3. Normalized absorption and fluorescence emission spectra of (A) PTTP-DC4/PTTP-DC4@L, (B) PTTP-DC6/PTTP-DC6@L, and (C) PTTP-DC8/PTTP-DC8@L. Spectra of free PTTP-DCns were recorded in different solvents, whereas PTTP-DCn@Ls were in 1×PBS.

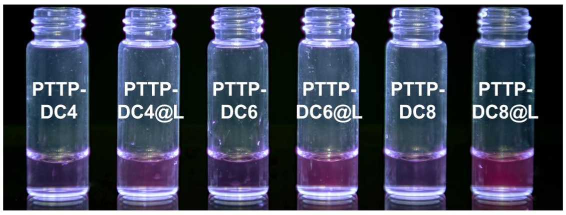

Supplementary Figure 4. Photograph of **PTTP-DCns** or **PTTP-DCn@Ls** in 1xPBS under the illumination of a 365 nm UV light. [**PTTP-DCns**] = 10 μM.

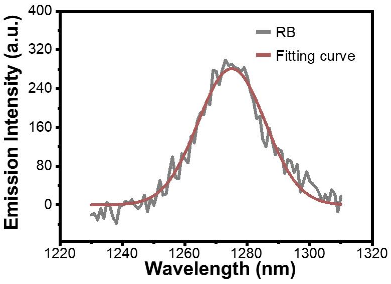

Supplementary Figure 5. The phosphorescence emission spectrum of $^1O_2$ generated by RB in deuterated PBS and relevant Gaussian fitting curve.

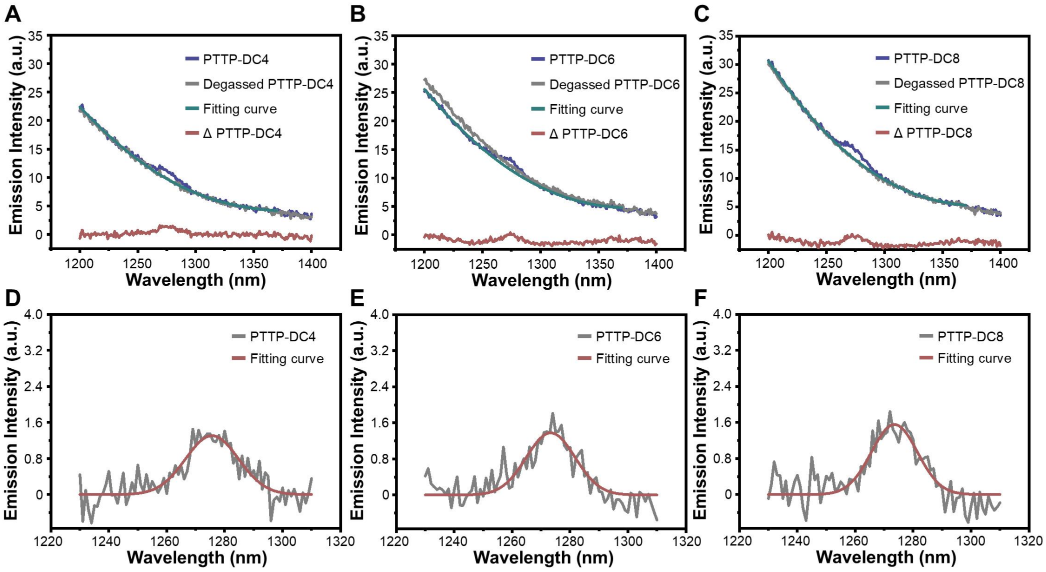

Supplementary Figure 6. (A-C) The phosphorescence emission spectra of **PTTP-DCns** in deuterated PBS before (navy curve) and after (gray curve) degassed with N_2. The polynomial fitting curve (green curve) was used as baseline to get the $^1O_2$ phosphorescence spectrum (red curve) after deduction; (D-F) The phosphorescence emission spectra of $^1O_2$ generated by **PTTP-DCns** in deuterated PBS and relevant Gaussian fitting curve.

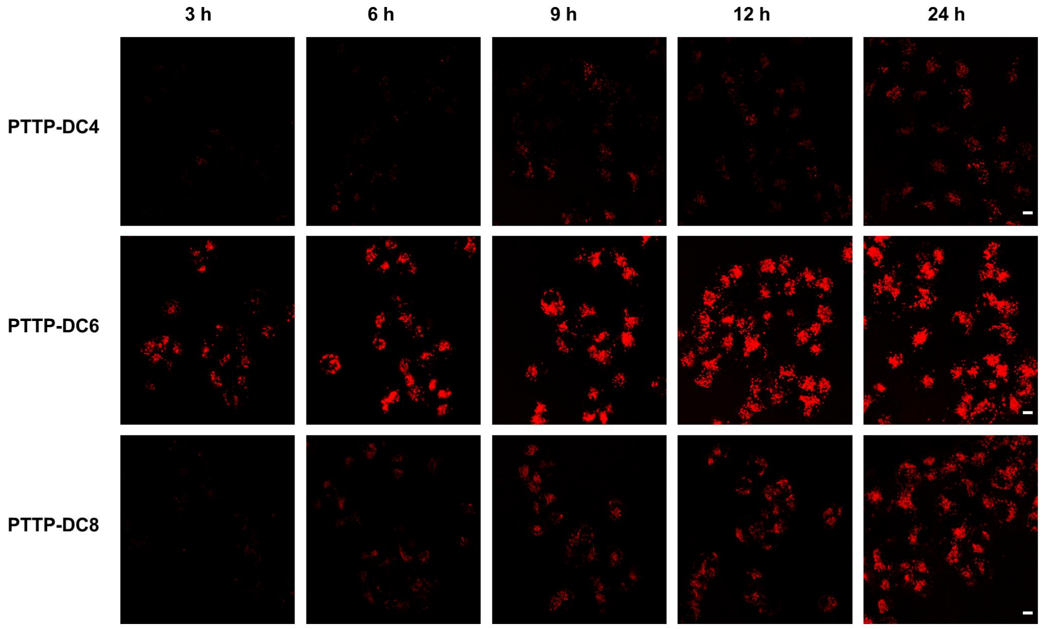

Supplementary Figure 7. Confocal images of MCF-7/ADR cells stained with 1 μM **PTTP-DCns** molecules in long periods. Scale bar: 10 μm.

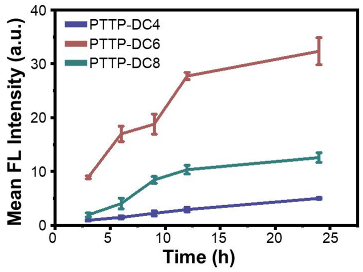

Supplementary Figure 8. Mean fluorescence intensities over time of **PTTP-DCns** in MCF-7/ADR cells. The intensities were taken from corresponding confocal images (Supplementary Figure 7). Data presented as mean ± SD.

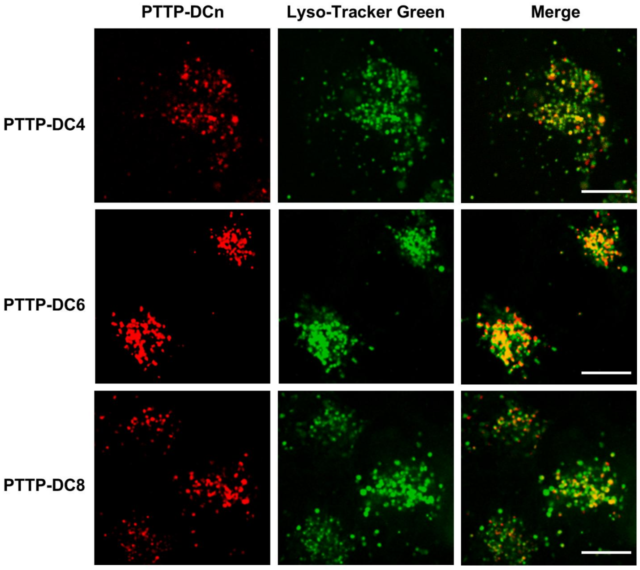

Supplementary Figure 9. Colocalization images of MCF-7/ADR cells after being incubated with **PTTP-DCns** for 24 h, and then stained by lysosome-specific dye LysoTracker Green for 1 h at 37 °C. The **PTTP-DCns** fluorescence channel (represented in red) was recorded using λ_{ex} = 552 nm and collecting the emission in the range of 660−740 nm. The LysoTracker fluorescence channel (represented in green) was recorded using λ_{ex} = 488 nm and collecting the emission in the range of 500−540 nm. [**PTTP-DCns**] = 1 μM, [LysoTracker Green] = 5 μM. Scale bar: 10 μm.

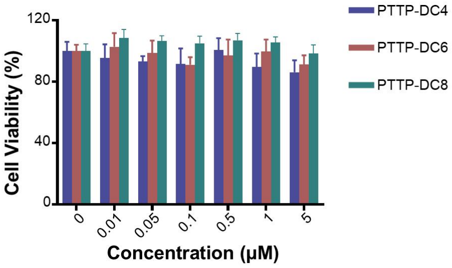

Supplementary Figure 10. Cell viabilities of HEK293 cells after being treated with different concentrations of **PTTP-DCns** for 48 h in dark. Data presented as mean ± SD.

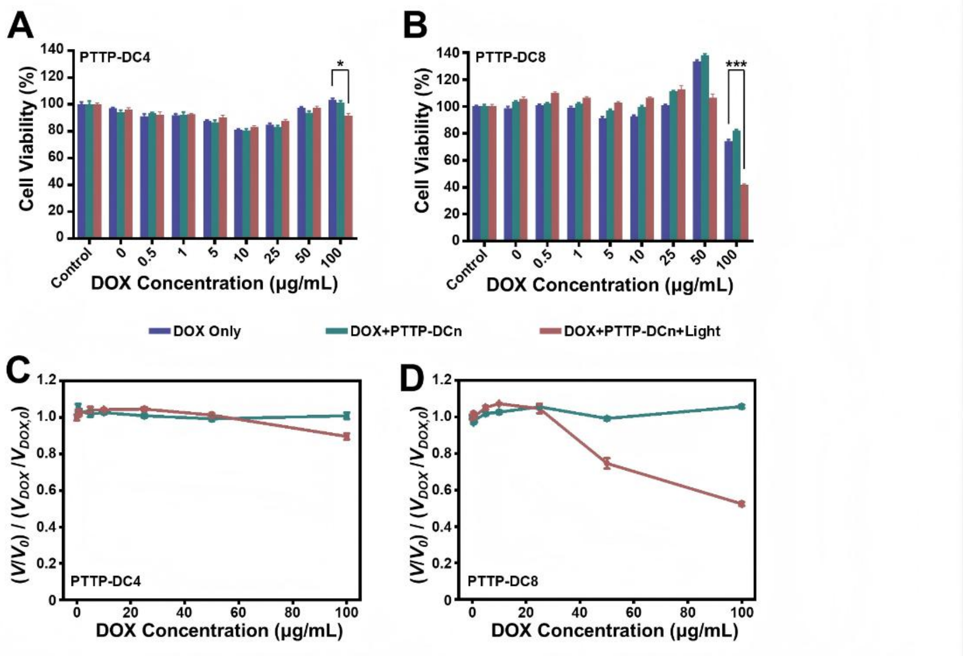

Supplementary Figure 11. Cytotoxicity of DOX to MCF-7/ADR cells after PTTP-DCns treatment under dark or light irradiation. The cells were incubated without or with 1 μM PTTP-DC4 (A) or PTTP-DC8 (B) for 24 h, irradiated with 525 nm light (0.2 mW cm^{-2}) or standing under dark for 30min, followed by 48 h incubation with DOX before MTT assay. (C-D) Relative viability rates of PTTP-DCns-treated group under dark or light irradiation referring to viabilities of control group treated with DOX only. V: the cell viability of combinations, V_{DOX}: the cell viability of DOX only. V_0,$V_{DOX,0}$: the cell viability in the 0 μM DOX group. Data presented as mean ± SD. One-way ANOVA with Tukey's test, *p < 0.05, ***p < 0.001.

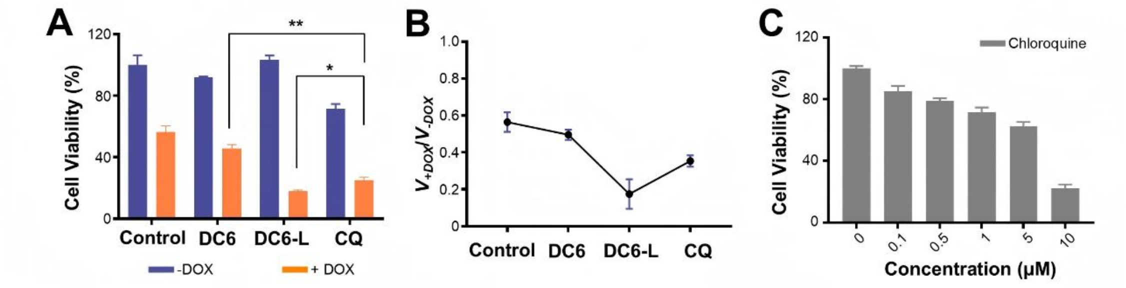

Supplementary Figure 12. (A) Cytotoxicity of DOX (100 μg mL^{-1}) to MCF-7/ADR cells after PTTP-DC6 (1 μM, 24 h) treatment under dark or light irradiation (525 nm, 0.2 mW cm^{-2}, 30 min) or chloroquine (CQ, 1 μM, 24 h) treatment (one-way ANOVA with Tukey's test, $*p < 0.05$, $**p < 0.01$); (B) Cell viability ratio in response to different lysosomal perturbation conditions. V_{+DOX}: the cell viability of DOX treatment., V_{-DOX}: the cell viability of DOX absence. (C) Cell viabilities of MCF-7/ADR cells after being treated with different concentrations of chloroquine for 24 h in dark. Data presented as mean ± SD.

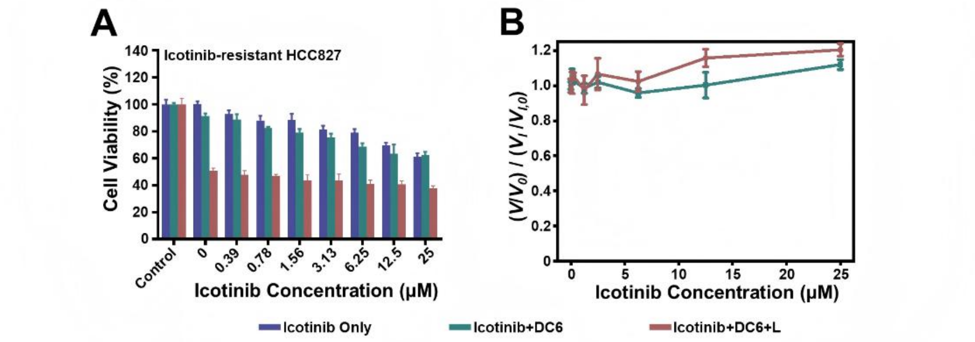

Supplementary Figure 13. (A) Cytotoxicity of icotinib to icotinib-resistant HCC827 cells after PTTP-DC6 (1 μM, 24 h) treatment under dark or light irradiation (525 nm, 0.2 mW cm^{-2}, 30 min); (B) Relative viability rates of PTTP-D6-treated group under dark or light irradiation referring to viabilities of control group treated with icotinib only. V: the cell viability of combinations, V_I: the cell viability of icotinib only, V_0,$V_{I,0}$: the cell viability in the 0 μM icotinib group. Data presented as mean ± SD.

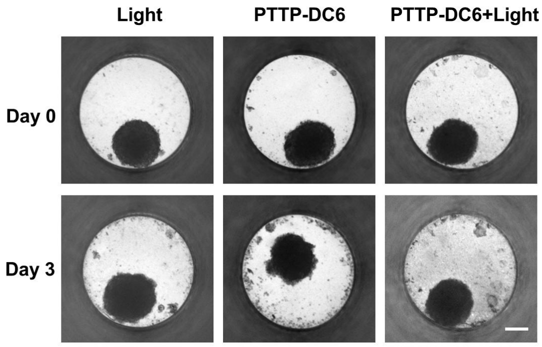

Supplementary Figure 14. Representative bright-field images of MCF-7/ADR cell spheroids with or without the treatment of **PTTP-DC6** (5 μM, 24 h) and the followed irradiation of a 525 nm LED light (1 mW cm-2, 30 min) on day 0 and day 3. Scale bar: 200 μm.

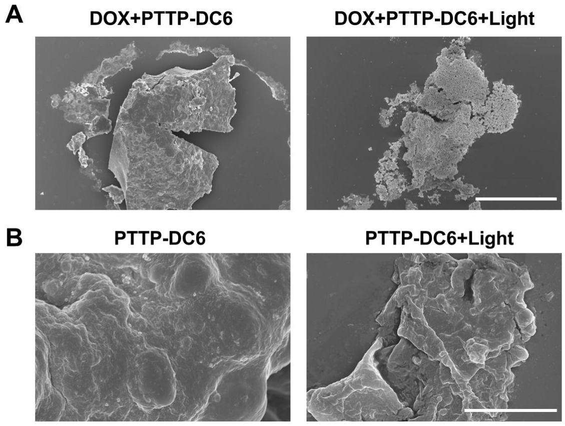

Supplementary Figure 15. SEM images of MCF-7/ADR cell spheroids of various treatment conditions on day 3 with different scale bar: (A) 100 μm and (B) 10 μm.

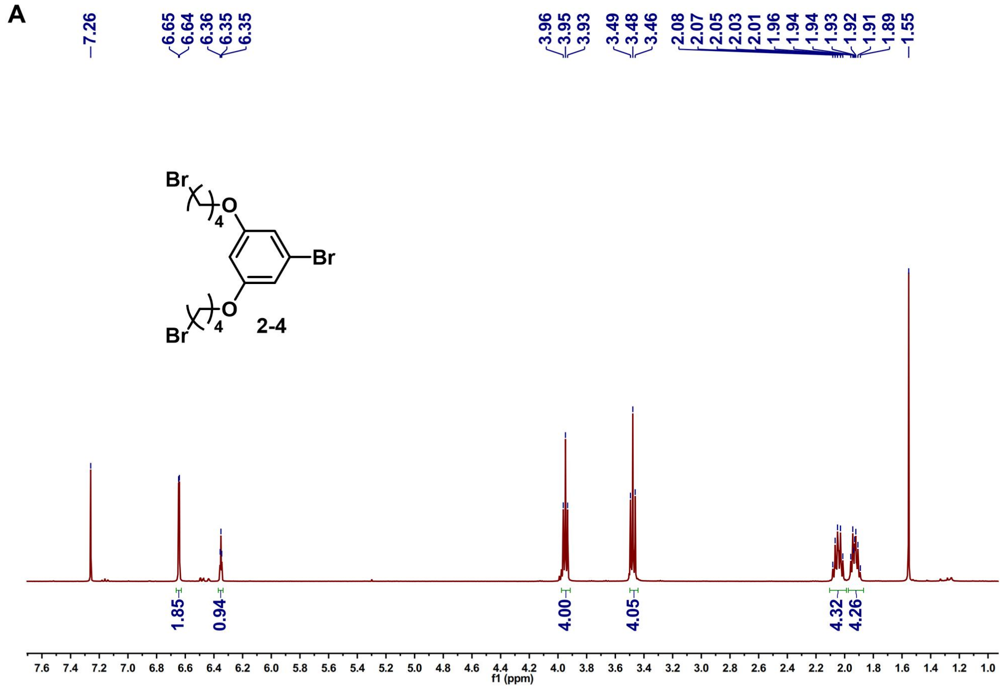

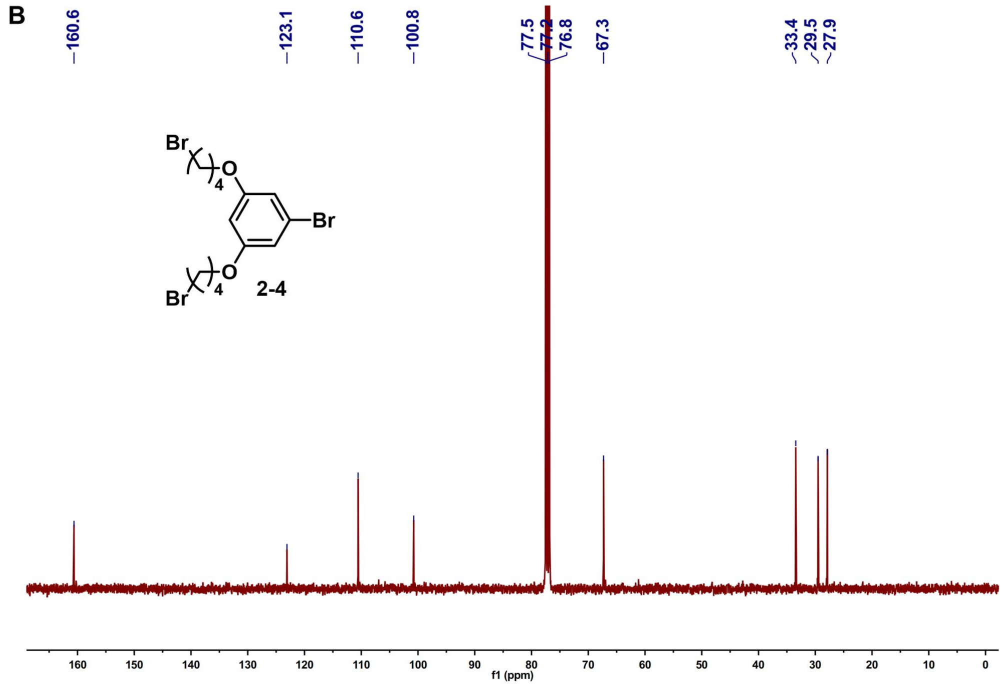

Supplementary Figure 16. (A) ^{1}H NMR and (B) ^{13}C NMR spectra of compound **2-4** in $CDCl_3$.

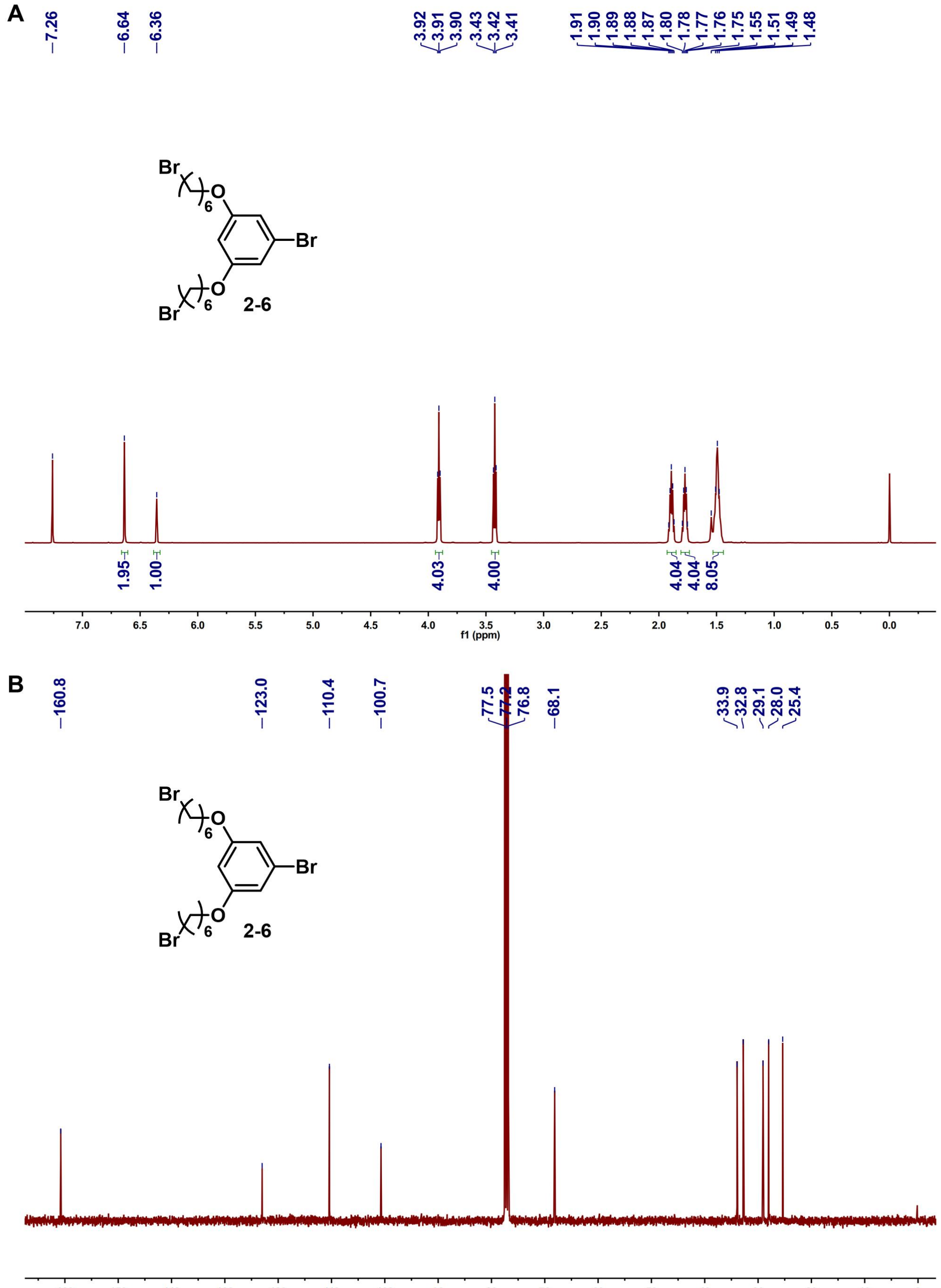

Supplementary Figure 17. (A) ^{1}H NMR and (B) ^{13}C NMR spectra of compound **2-6** in $CDCl_3$.

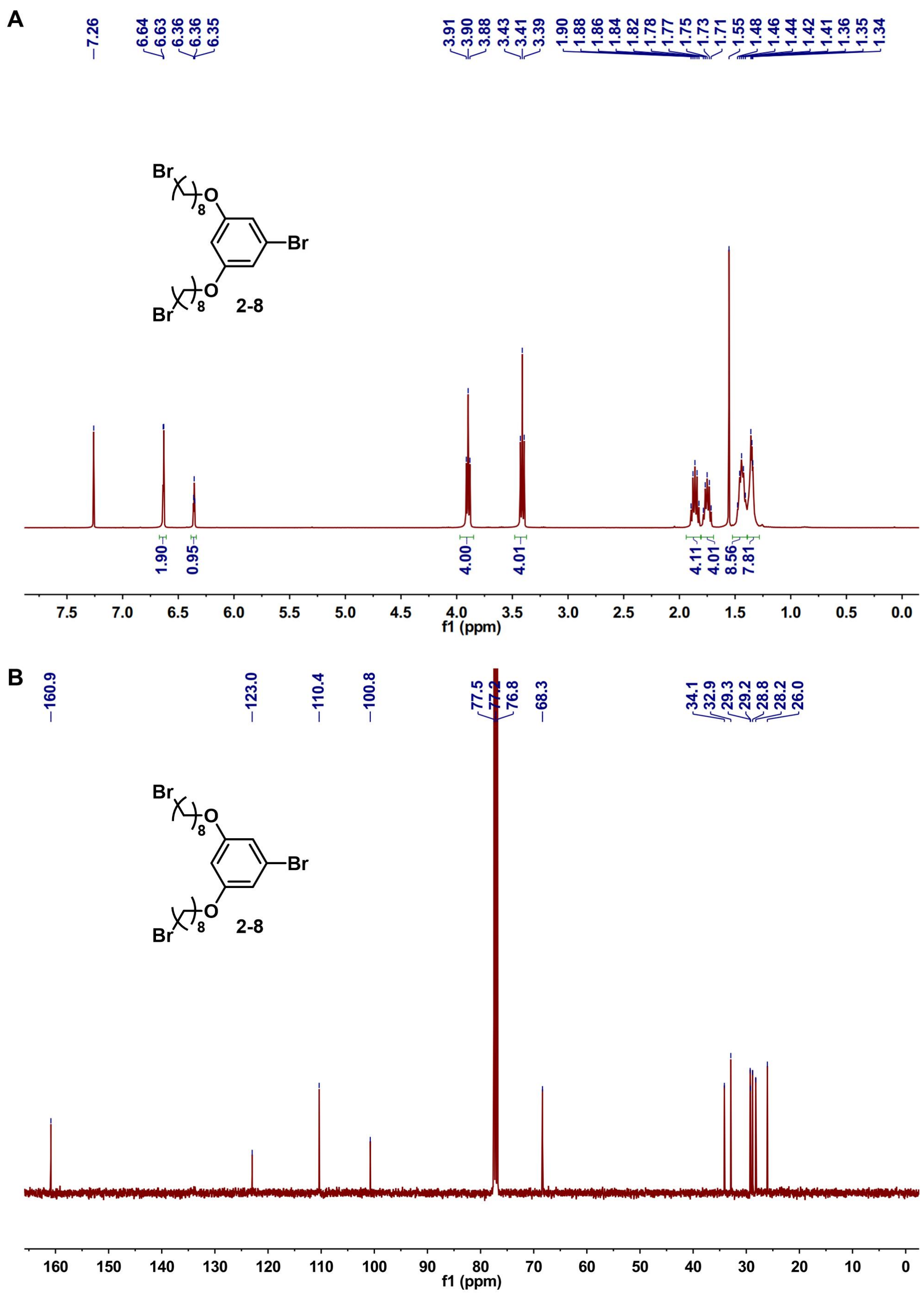

Supplementary Figure 18. (A) ^{1}H NMR and (B) ^{13}C NMR spectra of compound **2-8** in $CDCl_3$.

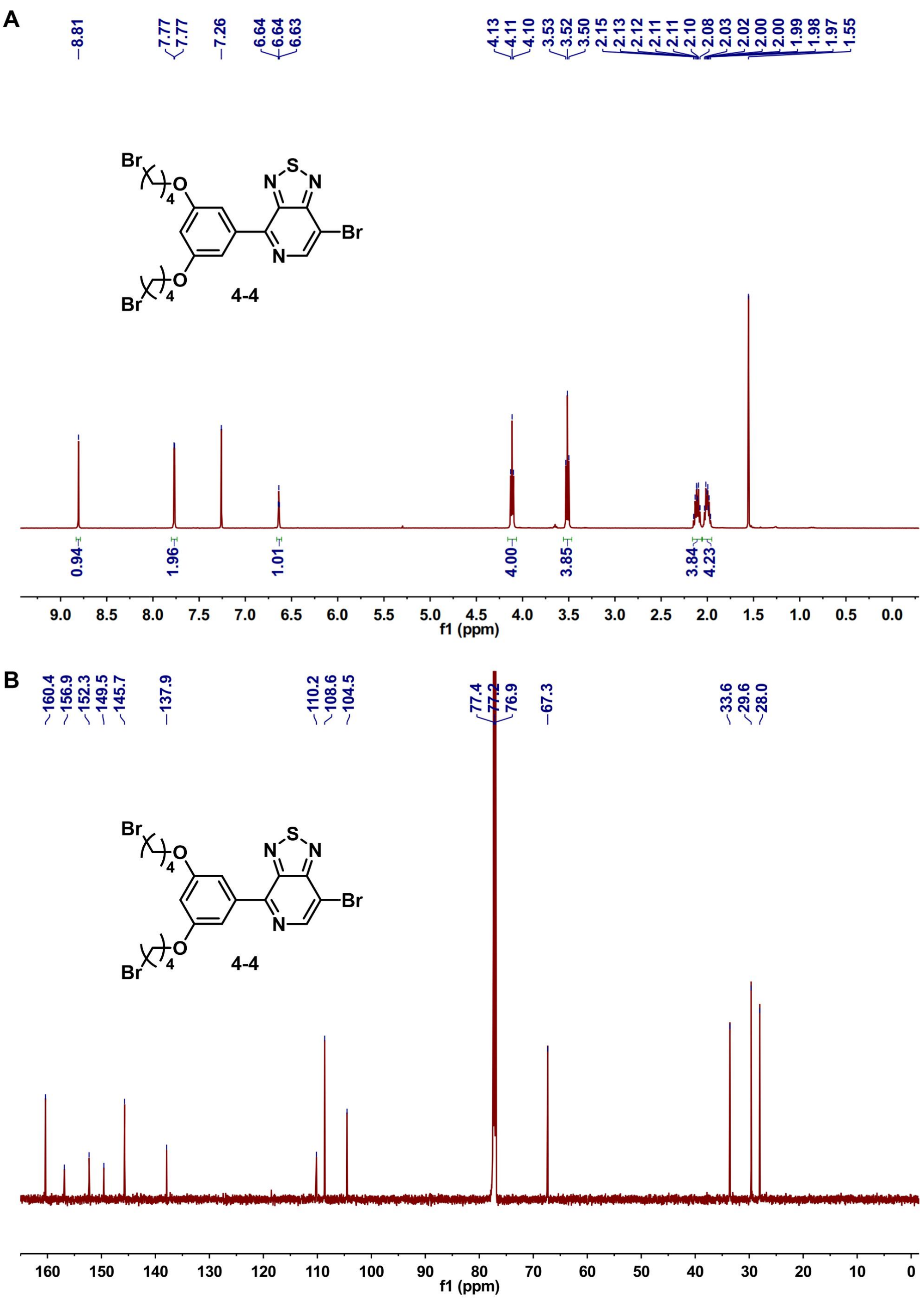

Supplementary Figure 19. (A) ^{1}H NMR and (B) ^{13}C NMR spectra of compound **4-4** in $CDCl_3$.

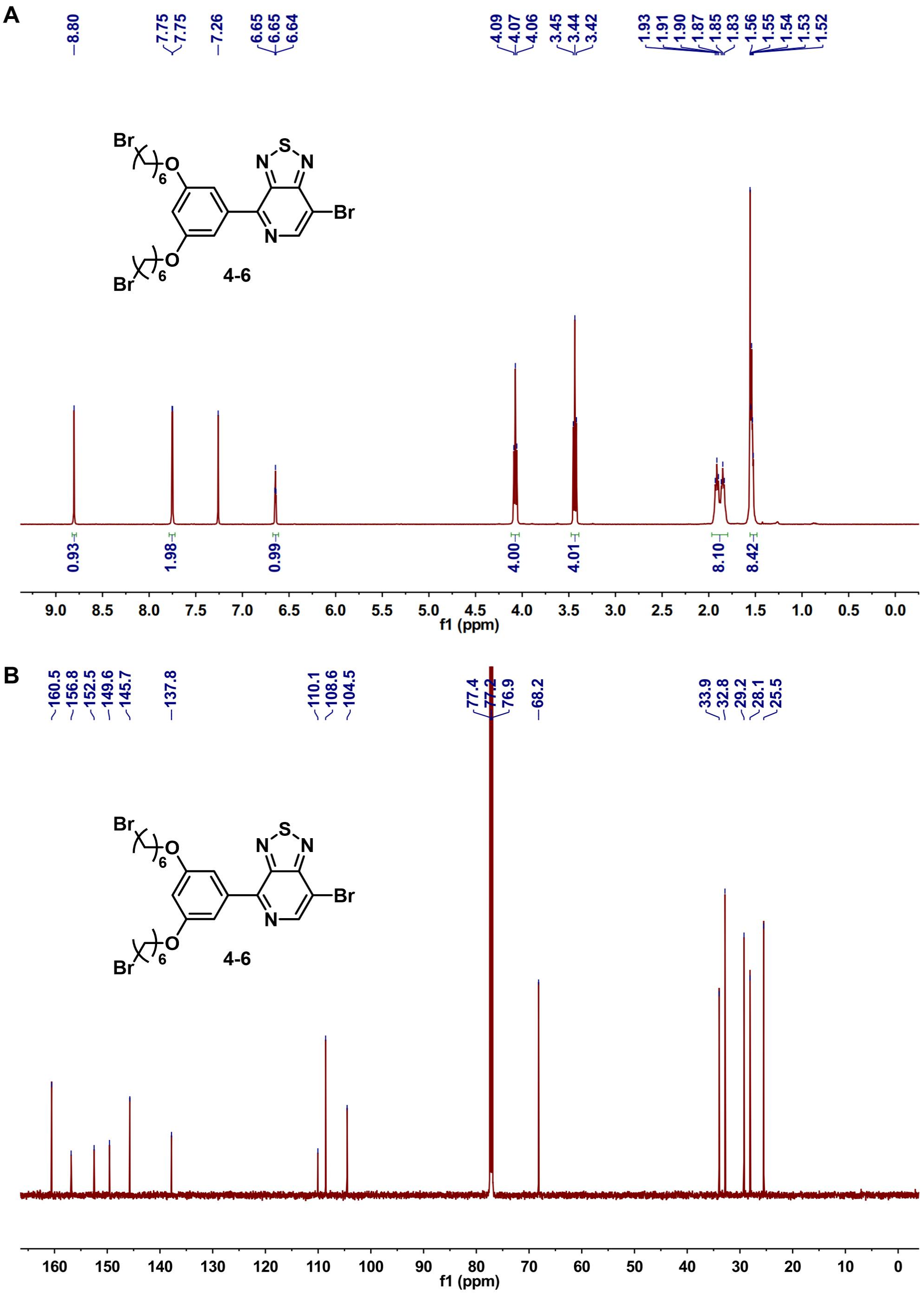

Supplementary Figure 20. (A) ^{1}H NMR and (B) ^{13}C NMR spectra of compound **4-6** in $CDCl_3$.

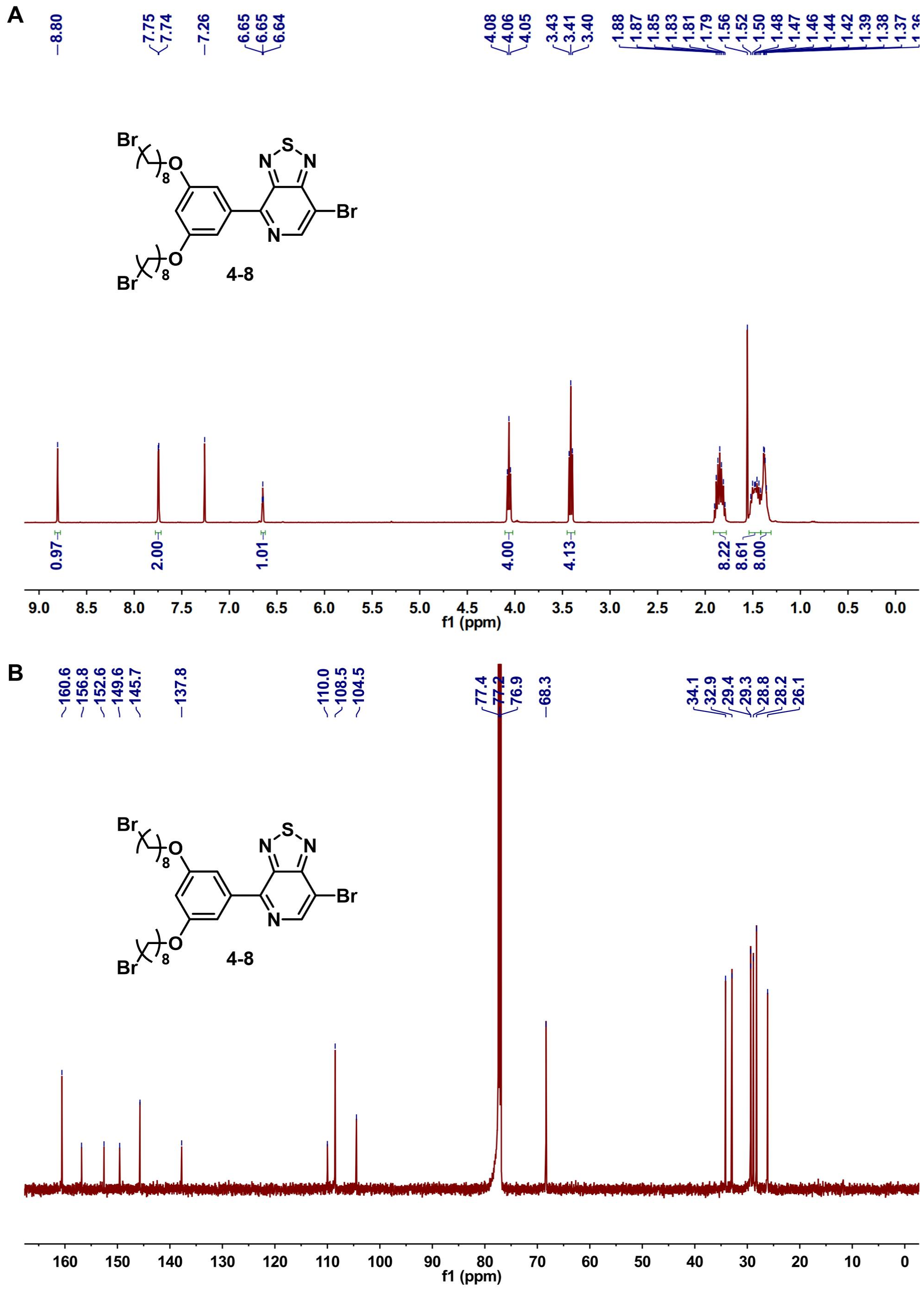

Supplementary Figure 21. (A) ^{1}H NMR and (B) ^{13}C NMR spectra of compound **4-8** in $CDCl_3$.

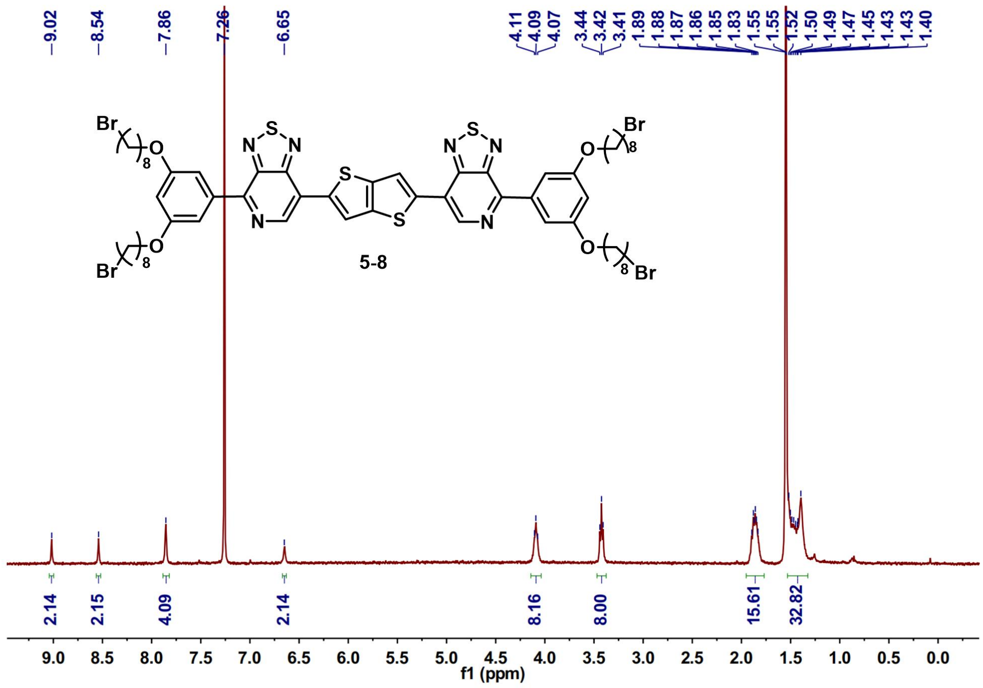

Supplementary Figure 22. ^{1}H NMR spectra of compound **5-8** in $CDCl_3$.

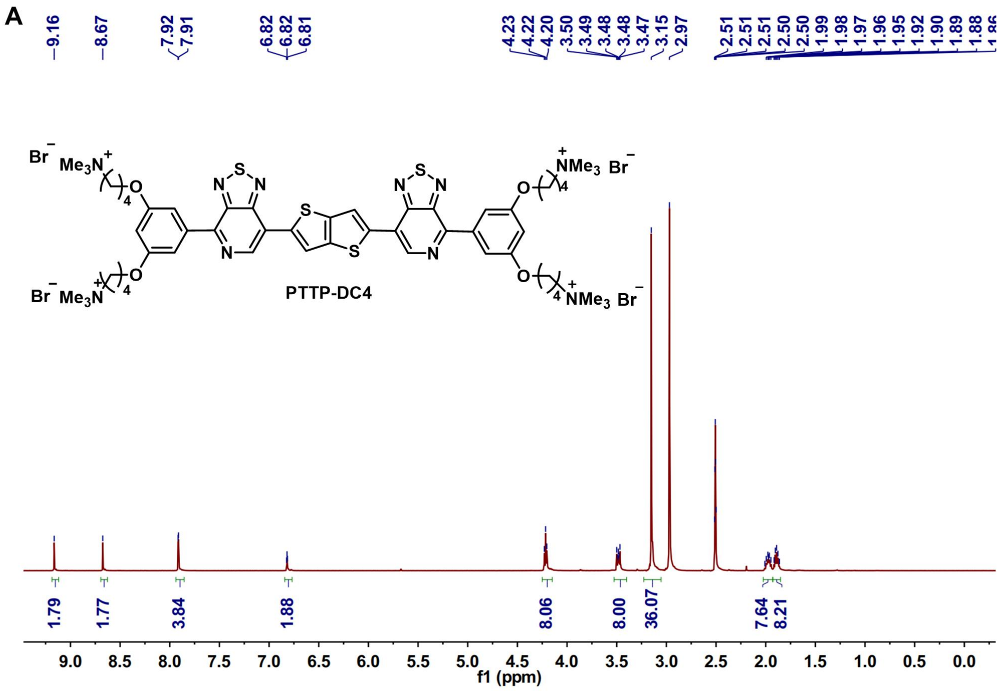

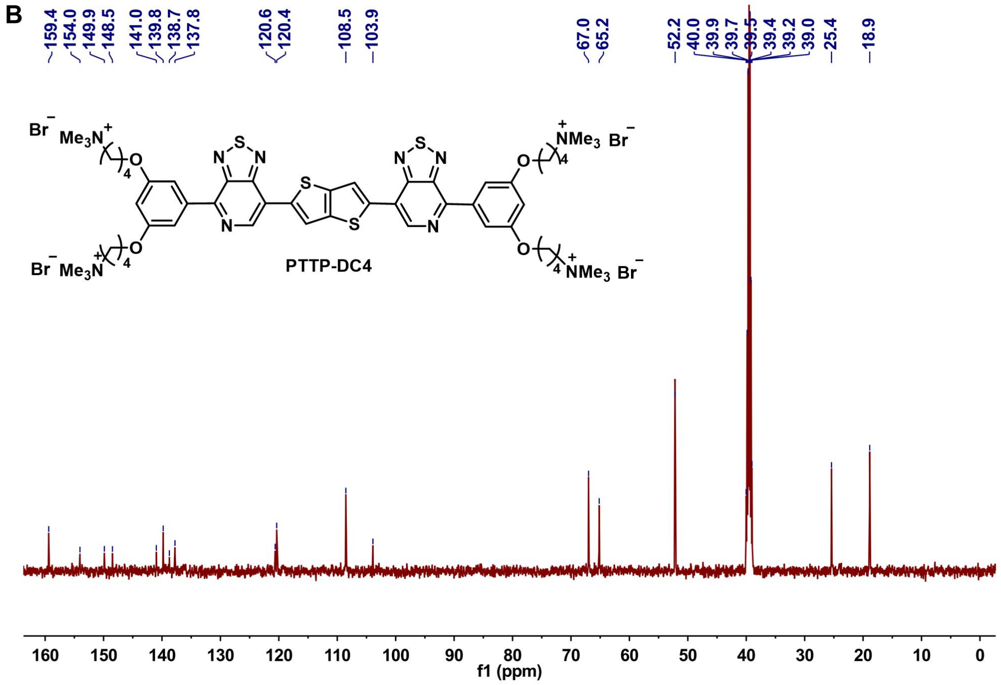

Supplementary Figure 23. (A) ^{1}H NMR and (B) ^{13}C NMR spectra of compound **PTTP-DC4** in DMSO-d6.

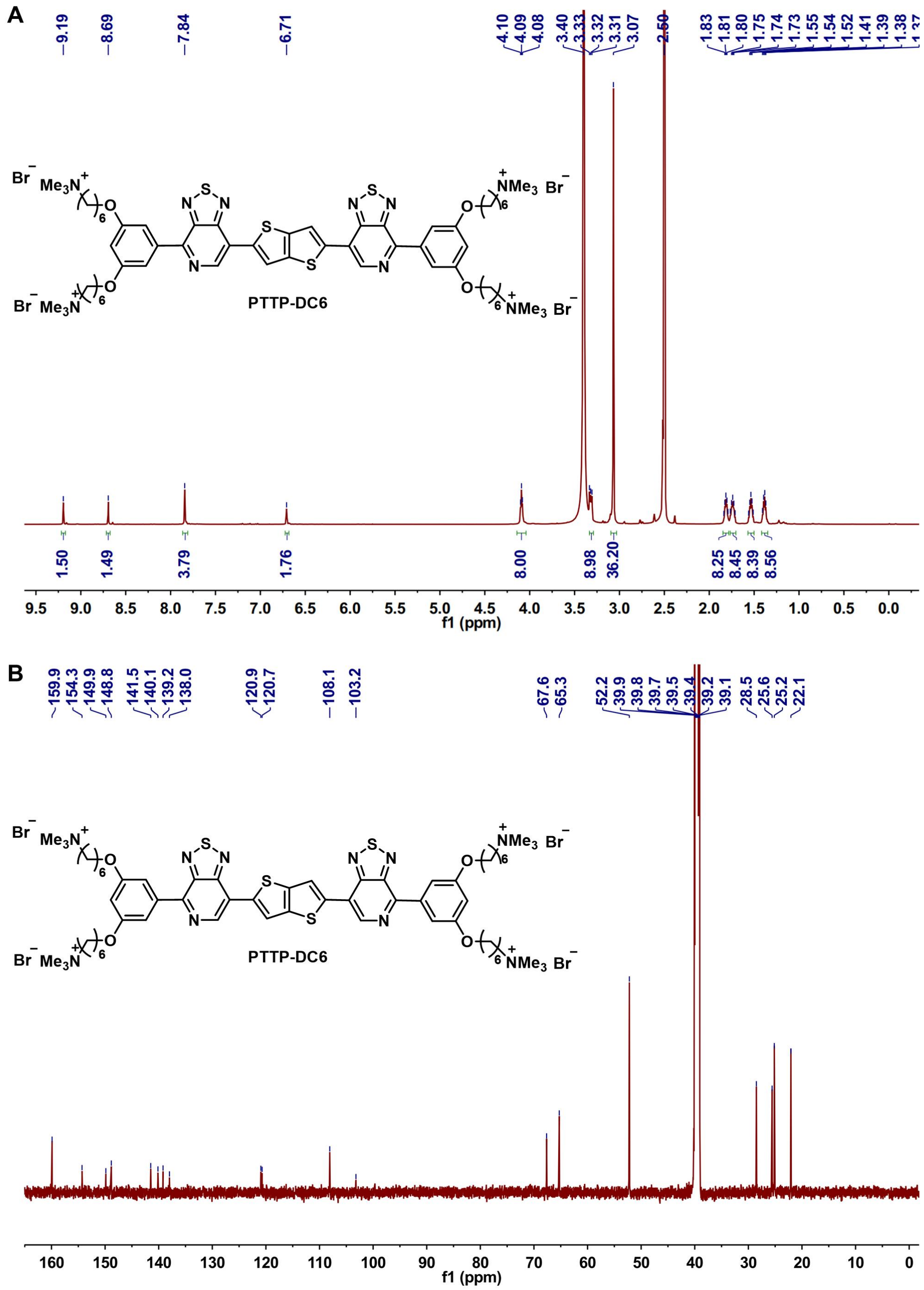

Supplementary Figure 24. (A) ^{1}H NMR and (B) ^{13}C NMR spectra of compound **PTTP-DC6** in DMSO-d6.

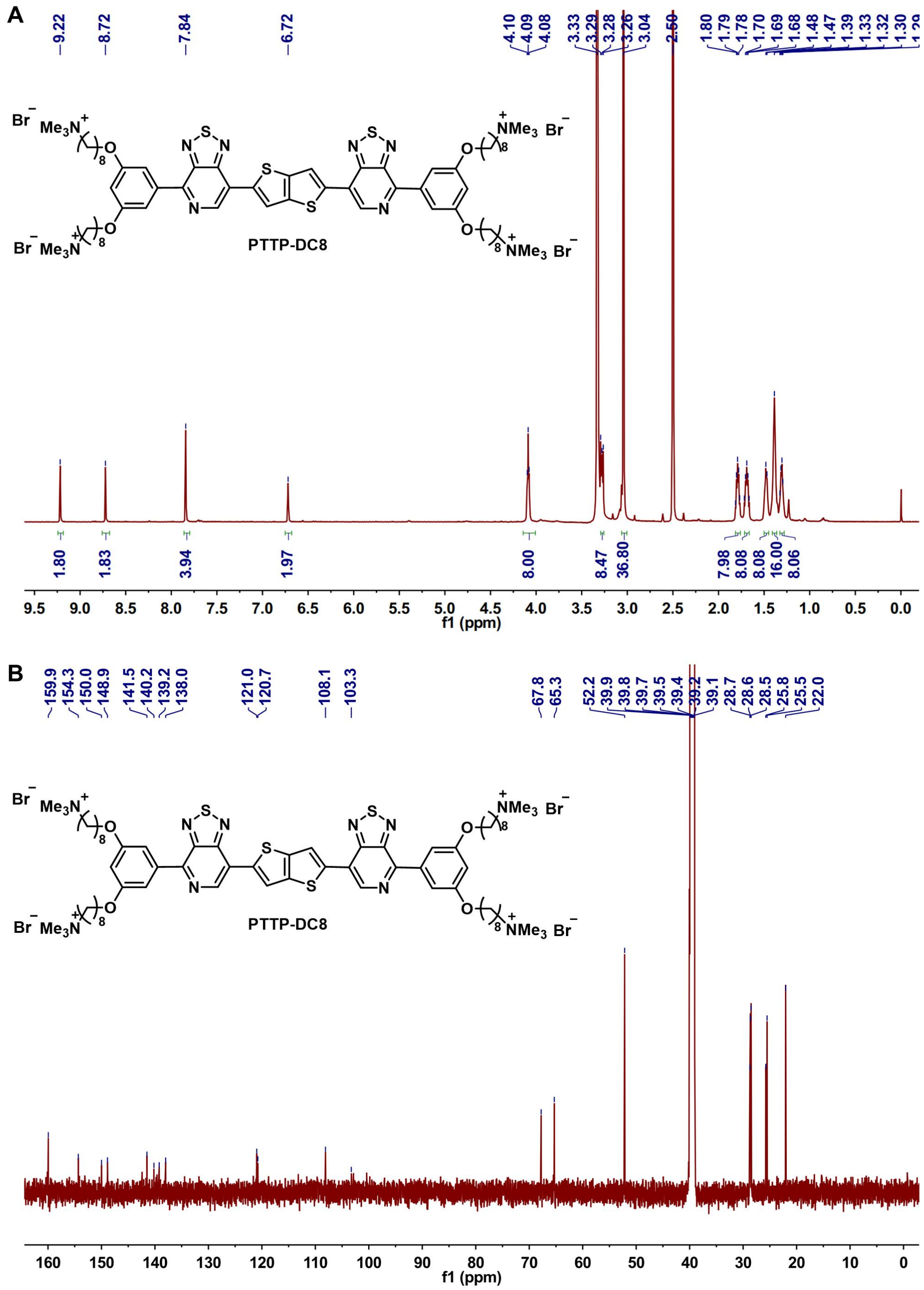

Supplementary Figure 25. (A) ^{1}H NMR and (B) ^{13}C NMR spectra of compound **PTTP-DC8** in DMSO-d6.
